# Discovery of a novel potent tubulin inhibitor through virtual screening and target validation for cancer chemotherapy

**DOI:** 10.1038/s41420-025-02679-3

**Published:** 2025-08-19

**Authors:** Peipei Shan, Kai-Lu Liu, Xiu Jiang, Guangzhao Zhou, Kongkai Zhu, Hua Zhang

**Affiliations:** 1 Institute of Translational Medicine, the Affiliated Hospital of Qingdao University, College of Medicine, Qingdao University, Qingdao, China; 2 School of Biological Science and Technology, University of Jinan, Jinan, China; 3 Qingdao Central Hospital, University of Health and Rehabilitation Sciences, Qingdao, China; 4 Department of Anesthesiology, The Affiliated Hospital of Qingdao University, Qingdao, China; 5 Advanced Medical Research Institute, Cheeloo College of Medicine, Shandong University, Jinan, China

**Keywords:** Cancer prevention, Virtual screening, Chemotherapy, Drug development

## Abstract

Microtubules, critical to diverse cellular processes, represent a clinically validated target for anticancer therapeutics. In this study, a virtual screening of the Specs library, consisting of 200,340 compounds, was conducted to target the taxane and colchicine binding sites on tubulin, resulting in the identification of 93 promising candidates for further analysis. Subsequent characterization revealed a nicotinic acid derivative (compound **89**) as a potent tubulin inhibitor, demonstrating significant anti-tumor efficacy in vitro and in vivo, with no observable toxicity at therapeutic doses in mice. Notably, compound **89** also exhibited robust antitumor activity in patient-derived organoids. Mechanistic studies, including EBI competitive binding assays and molecular docking, confirmed its inhibition toward tubulin polymerization via selective binding to the colchicine site. Furthermore, compound **89** disrupted tubulin assembly dynamics through modulation of the PI3K/Akt signaling pathway. This work presents a novel tubulin-inhibiting scaffold with potential for advancing next-generation microtubule-targeted chemotherapies.

## Introduction

Microtubules play crucial roles in many cellular events, comprising formation of spindles, intracellular signaling transduction and substance transportation, as well as regulation of cell motility, division and apoptosis [1–3]. Its polymeric structures compose of the heterodimers of α and β-tubulin, which is a highly dynamic process characterized by the rapid cycles of polymerization/depolymerization through the addition or removal of tubulin dimers [4, 5]. The dynamics of microtubule assembly is precisely modulated to mediate the aforementioned cellular events, and any disturbance or interference on this process would lead to G_2_/M phase arrest of cells, eventually causing cell death [6–8]. Hence, the exploration of microtubule-targeting agents (MTAs) to perturb the dynamic stability of microtubule assembly represents an important strategy in antitumor therapy [9–11].

Given their important roles in regulating cellular processes, microtubules had been recognized as an ideal target for the development of antitumor medications since half century ago [12, 13]. Based on the different modes of action in modulating microtubule dynamics, MTAs can be primarily classified into microtubule-stabilizing agents and microtubule-destabilizing agents: the former enhance tubulin polymerization, while the latter inhibit tubulin polymerization [2]. During the last several decades, numerous chemical compounds have been identified as promising MTAs with potent antitumor effects [14, 15]. At present, some MTAs such as taxol (paclitaxel), vinblastine and vincristine are still applied to clinical treatment especially for advanced cancers [13, 16]. Despite the great success of MTAs in anticancer drug research, there are still many challenges, including side effects and drug resistance, yet to be solved [17, 18]. Thus, continuing efforts in the discovery and development of more MTAs with new chemical scaffolds, improved activities and reduced side effects are still of high demand.

Colchicine-site tubulin inhibitors are well-known molecules that bind to an interface region of the α and β-tubulin subunits [12]. Recently, inhibitors targeting the colchicine site have received tremendous attention due to their superior therapeutic potential (compared with other binding-site inhibitors), such as good water solubility, enhanced ability to inhibit angiogenesis and overcome multidrug resistance, and lower side effects [19, 20]. Several molecules binding to the colchicine-site are currently in clinical trials, including CA-4P, ABT-751, OXi4503, VERU-111 and BNC105P [20–23], but none of them have been approved for clinical use. Hence, more endeavors are still required to uncover new chemical frameworks in order to address the current limitations.

In a recent work, we described the identification of a novel type of natural inhibitor of tubulin from *Morinda officinalis* [24]. As a continuation of the previous efforts for novel small molecules targeting microtubule, we lately carried out a virtual screening on the commercial Specs library containing >200 thousand compounds, which was reworded with the discovery of two hit molecules **82** and **89** showing significant cytostatic activity against human Hela (cervical) and HCT116 (colonic) tumor cells. Subsequent antineoplastic evaluation and mechanistical exploration on the more active **89** identified it as a potent colchicine-site inhibitor that promoted the polymerization of tubulin. Compound **89** could interfere with the assembly of microtubules by inhibiting PI3K/Akt signaling pathway, exerted good in vitro and in vivo antitumor effects, and more excitingly inhibited the growth of patient-derived organoids. Hence, details of the aforementioned investigations will be described below.

## Results

### Identification of 89 as a potential tubulin inhibitor with antiproliferative activity

**Fig. 1:**
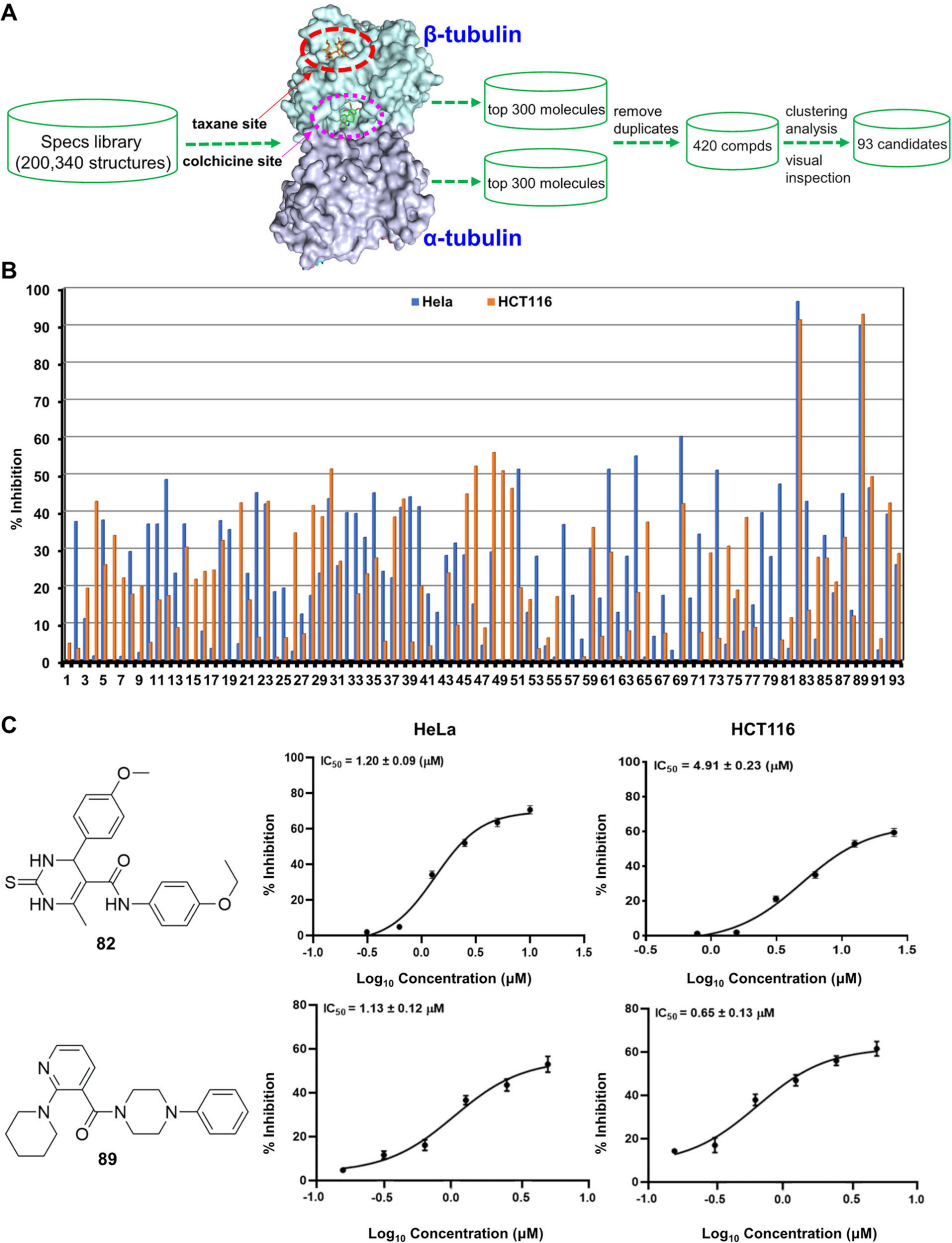
Virtual screening for MTAs identified compound 89 as a new potential anti-tumor small molecule. **A** High-throughput virtual screening workflow for new tubulin inhibitors. **B** HeLa and HCT116 cells were treated with the indicated compounds, and MTS assay was performed after 24 h. **C** The chemical structures of compounds **82** and **89**, and their IC_50_ values against HeLa and HCT116 cells.

MTAs are usually categorized into two main groups, namely, microtubule-stabilizing agents and microtubule-destabilizing agents [25]. Among the seven known binding sites so far [26, 27], the classical taxane site and colchicine site, representative for the aforementioned two groups of MTAs, respectively, were chosen for the virtual screening in the current work, and the whole process was shown in Fig. 1A. The commercially available Specs library with 200,340 synthetic molecules (https://www.specs.net/) was applied to the molecular docking using Glide 5.5 program [28]. Top 300 structures for each binding site were first selected according to their docking scores, and a total of 420 compounds were obtained after the removal of duplicate molecules. Finally, 93 candidates were picked out for further study based on clustering analysis and visual inspection.

The 93 compounds identified in the aforementioned computational screening were then purchased (the original Specs codes of these molecules that can be used to check their structures were provided in Supplementary Table S1), and their antiproliferative activities against human Hela (cervical) and HCT116 (colonic) tumor cell lines were then tested at 50 μM. As shown in Fig. 1B, compounds **82** and **89** (Fig. 1C) displayed significant growth inhibition toward both cells (>90% inhibitory rate), while other compounds only showed weak or no cytostatic effect (mostly <50% inhibitory rate). The IC_50_ values of **82** and **89** were further acquired, revealing a better activity for **89** than **82** against both cell lines (Fig. 1C).

To check if there are superior molecules in the Specs library, a retrieval for structural analogs of **89** was conducted, and 17 additional compounds were picked out, purchased and tested for their antiproliferative effect toward the aforementioned two cell lines (Supplementary Table S2). The structure-activity relationship of all the tested molecules was then briefly discussed below. Compared with **89**, replacement of the phenyl unit by a hydroxyethyl group in **A2** caused the loss of inhibitory activity, while reduction of the carbonyl group to a methylene in **A5** also resulted in a decline in activity. Interestingly, a growth promoting effect was observed when the phenylpiperazine moiety was replaced by a piperidine unit in **A3**, whereas both the growth promoting and inhibitory effect disappeared upon further substitution of this piperidine unit by a morpholine fragment in **A4**. It was surprising to find that the cytostatic activity partially recovered by replacing the 2-piperidinyl group (of the pyridine ring) in **A4** with a hydroxyethylpiperazinyl unit in **A6**. Nevertheless, compounds **A1** and **A7** with piperidine and pyrrolidine moieties replacing the morpholine unit in **A6**, respectively, did not show obvious inhibition against both cell lines. In addition to the above-mentioned molecules, only compound **B3** with 2-isopropylpiperazinyl-5-pyrrolidinylcarbonyl substitution on the pyridine ring, among others bearing more structural variations, displayed moderate antiproliferative activity. Therefore, compound **89** appeared to be a potential lead compound as tubulin inhibitor and was thus chosen for the subsequent antitumor evaluation and mechanistical investigations.

### Compound **89** inhibited the proliferation, invasion and migration of tumor cells

**Fig. 2:**
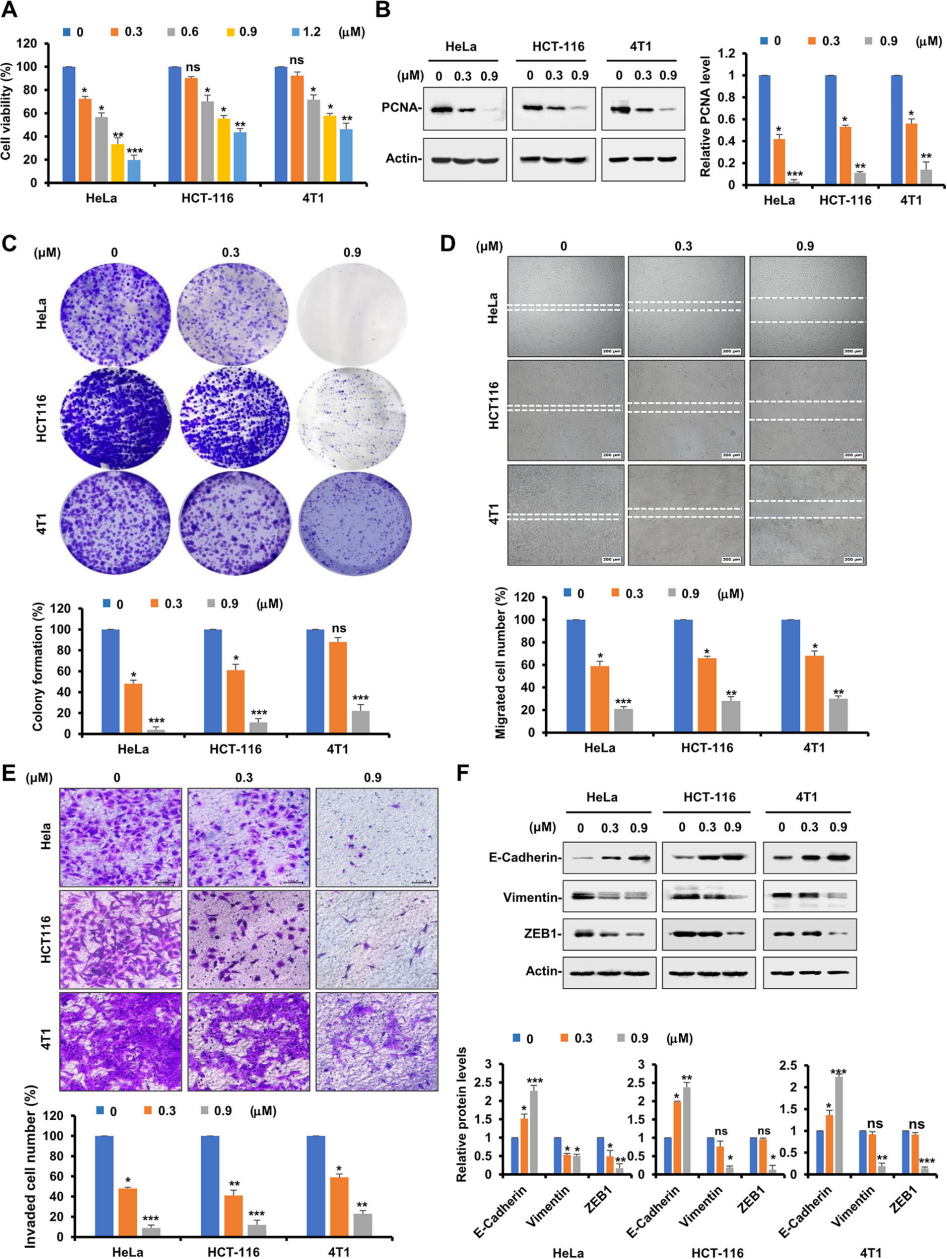
Compound 89 inhibited the proliferation, invasion and migration of tumor cells. **A** HeLa, HCT116 and 4T1 cells were treated with increasing concentrations of **89**, and MTS assay was performed after 24 h. The bars indicate mean ± SD (*n* = 3). **B** HeLa, HCT116 and 4T1 cells were treated with indicated concentrations of **89**, and the expression of PCNA was detected by Western blot assay. Actin was used as loading control. **C** HeLa, HCT116 and 4T1 cells were seeded in 6-well plates, and after 12 h, cells were treated with indicated concentrations of **89**. On day 10, the number of colonies was counted in experiments repeated three times. Results represent the average of three replications. **D** HeLa, HCT116 and 4T1 cells were seeded in 6-well plates. A “wound” was created after the cells grew into full confluence, and different concentrations of **89** were added. Images were taken after 12 h of incubation at 37 °C. **E** HeLa, HCT116 and 4T1 cells were re-suspended in serum-free medium and seeded into the upper side of the transwell insert pre-coated with Matrigel. Increasing concentrations of **89** were added to both chambers, and images were obtained after 12 h of incubation. Bars represent mean ± SD of three independent experiments. **F** HeLa, HCT116 and 4T1 cells were treated with indicated concentrations of **89**, and the expression of EMT-related proteins was detected by Western blot assay with indicated antibodies. Bars represent mean ± SD from three independent experiments.

The antiproliferative effect of **89** was further evaluated in Hela, HCT116 and 4T1 cells by MTS assay. As can be seen in Fig. 2A, **89** significantly reduced the viability of the three cancer cells in a dose-dependent manner. In addition, **89** also showed antitumor activity on other tumor cell lines of different origin (A549, H1299, MDA-MB231), indicating that **89** could be a broad-spectrum antitumor agent like other tubulin inhibitors (Supplementary Fig. S1). Moreover, immunoblotting assay revealed a substantial downregulation of the PCNA protein (proliferating cell nuclear antigen) following treatment with **89** (Fig. 2B). Colonial formation effectively mimics the in vivo pathological process of tumor progression, so the clonogenic ability of the three cell lines was then analyzed after treatment with **89**. As shown in Fig. 2C, **89** markedly suppressed the colony formation of tumor cells in a dose-relying mode.

Next, the anti-migratory and anti-invasive effects of **89** toward the three cell lines were then evaluated. The data shown in Fig. 2D clearly indicated that the wound closure of the three cell lines was all markedly blocked by co-incubation with **89** in the wound healing experiment. Meanwhile, representative pictures and cell counts in Fig. 2E demonstrated that the invasion of tumor cells was remarkably prevented by treatment of **89** in the Transwell assay. EMT (Epithelial-mesenchymal transition) is regarded as a crucial mechanism governing the early stages of tumor metastasis [29], and the protein levels of relevant biomarkers were subsequently checked after administration of **89**. The data of Fig. 2F illustrated that **89** could significantly increase the expression of E-cadherin (epithelial indicator) while decrease that of ZEB1 (zinc-finger E-box binding homeobox 1) and vimentin (mesenchymal markers). Together, these findings collectively suggest that **89** exhibits strong inhibitory effects on tumor metastasis in vitro.

### Compound 89 induced cycle arrest and apoptosis in tumor cells

**Fig. 3:**
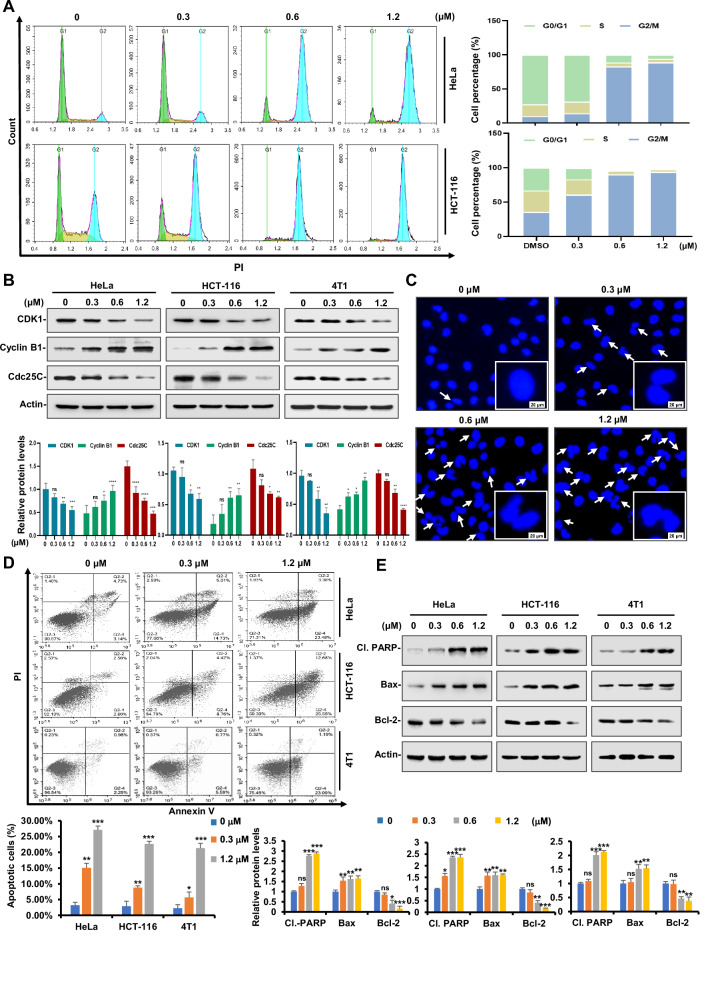
Compound 89 induced cycle arrest and apoptosis in tumor cells. **A** HeLa and HCT116 cells were treated with indicated concentrations of **89** and co-incubated for 24 h. Cell population distribution was determined following PI staining and further analyzed by flow cytometry. Bars represent mean ± SD of three independent experiments. **B** HeLa, HCT116 and 4T1 cells were treated with indicated concentrations of **89** and the expression of cell cycle-related proteins was detected by Western immunoblotting assay with the indicated antibodies. **C** HeLa cells were treated with **89** at the indicated doses. Nuclei were stained with DAPI (blue) by immunofluorescence staining. Scale bar, 20 μm. **D** HeLa, HCT116 and 4T1 cells were left untreated or treated with **89** at the indicated doses for 24 h. Apoptotic cells were labeled with annexin V and PI and analyzed by flow cytometry. Bars represent mean ± SD of three independent experiments. **E** HeLa, HCT116 and 4T1 cells were treated with indicated concentrations of **89** and the expression of cleaved-PARP, Bax and Bcl-2 were detected by Western immunoblotting assay with the indicated antibodies. Protein expressions were quantitated by densitometry and normalized against that of actin. Bars represent mean ± SD of three independent experiments.

Cell cycle arrest and apoptosis are the primary mechanisms through which most anticancer drugs eliminate tumor cells. Flow cytometric experiment was then performed to investigate whether **89** could induce cell cycle arrest and apoptosis in tumor cells. Hela and HCT116 cells after exposure to **89** were first examined for their cycle distributions, and as illustrated in Fig. 3A, treatment of **89** caused a significant G_2_/M phase arrest in both cell lines. **89** also induced G_2_/M phase arrest in 4T1 cell lines (Supplementary Fig. S2). Subsequently, an immunoblotting assay was conducted to evaluate the impact of **89** on the expression of CDK1, Cyclin B1 and Cdc25c (G_2_/M phase related proteins). The results in Fig. 3B showed that **89** upregulated the expression of cyclin B1 while downregulated those of CDK1 and Cdc25c. In addition, the effect of **89** on the morphology of HeLa cells was further examined through Hoechst 33342 staining (Fig. 3C), which clearly revealed an accumulation of M-phase cells.

Next, Annexin V-FITC/PI analysis demonstrated that **89** remarkably boosted the proportion of apoptotic cells in all three tested cell lines (Fig. 3D). Afterwards, immunoblotting assay demonstrated that the expression of Cl. PARP (cleaved PARP), which is a biomarker of apoptosis, gradually enhanced with the treatment of escalating doses of **89** (Fig. 3E), while those of Bax and Bcl-2 as pro-apoptosis and anti-apoptosis cytokines were up and down regulated, respectively.

### Compound 89 served as a tubulin polymerization inhibitor of the colchicine site

**Fig. 4:**
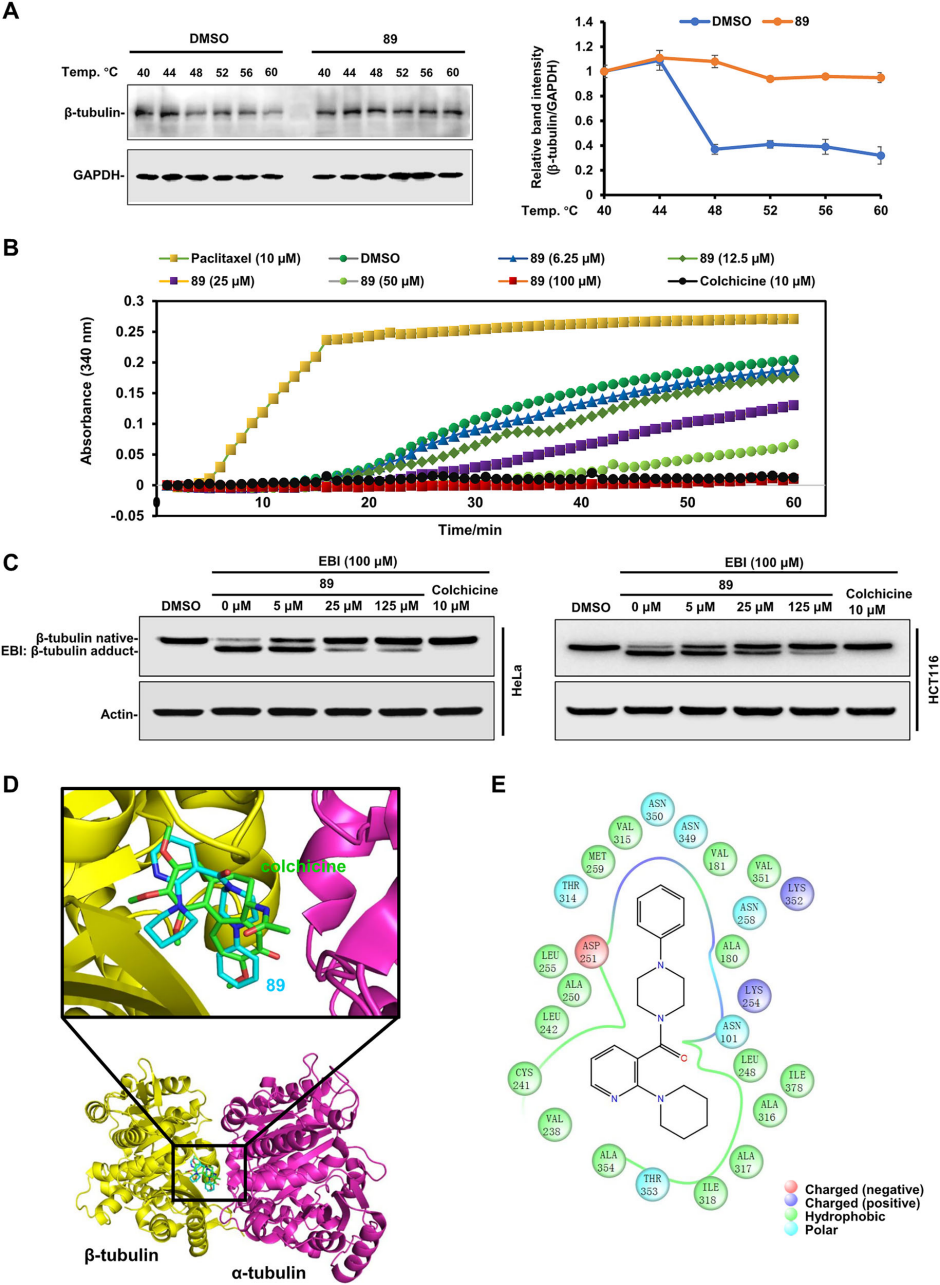
Compound 89 was identified as a potent tubulin polymerization inhibitor binding to the colchicine site. **A** HeLa cells were lysed using liquid nitrogen and three repeated cycles of freeze-thaw, and the cell lysate was treated with **89** (10 μM) or DMSO for 30 min at r.t. The cell suspension was heated for 3 min to 40, 44, 48, 52, 56 and 60 °C, cooled at 25 °C for 3 min, and then centrifuged at 20,000 × *g* for 30 min. Finally, the supernatant was collected for Western blot analysis. **B** In vitro tubulin polymerization assay was performed. β-tubulin was exposed to DMSO, colchicine (10 µM), paclitaxel (10 µM) or the indicated concentrations of **89**. GTP was added to initiate the reaction. The tubulin polymerization rate was monitored for 60 min at 37 °C and the absorbance at 340 nm was measured. **C** Six-well plates were seeded with HeLa or HCT116 cells (2 × 10^5^) for 24 h. The tumor cells were incubated with **89**, colchicine or DMSO for 2 h and afterward treated with EBI (100 μM) for 1.5 h. Cells were finally harvested and lysed, and the cell extracts were used for Western blotting analysis. **D** Cut-away view of the ligand-binding pocket at the colchicine site in the docking complex of **89** with tubulin. **E** Diagrammatic illustration of the interactions between tubulin protein and **89**.

To validate whether compound **89** directly targeted microtubule, CETSA (cellular thermal shift assay) was performed. The protein level of β-tubulin in the DMSO-treated group (as a control) (Fig. 4A) declined in pace with the rising temperatures, indicating a protein degradation, while that in the **89**-treated group remained relative stable, which suggested the direct binding of **89** to β-tubulin.

To further check which type of MTA **89** is (stabilizing or destabilizing agent), the tubulin polymerization experiment was conducted, using colchicine (destabilizer) and paclitaxel (stabilizer) as reference compounds. The curves in Fig. 4B indicated that paclitaxel rapidly promoted the polymerization of tubulin as anticipated, while in the presence of colchicine the tubulin assembly was severely impeded. Moreover, compound **89** exerted a similar effect to colchicine, disrupting the tubulin polymerization in a concentration-relying mode. These observations indicated that **89** functioned as a tubulin polymerization inhibitor (or microtubule destabilizer).

Since compound **89** was screened out based on taxane and colchicine sites and the aforementioned assay confirmed its inhibitory effect on the polymerization of tubulin, it was thus assumed to be a colchicine-site inhibitor. Then, the EBI (*N*,*N*′-ethylenebis) competition assay was performed to verify this hypothesis. As shown in Fig. 4C, only β-tubulin was detected in the blank and positive control groups, and both β-tubulin and EBI/β-tubulin adducts were detected in co-treatment groups of EBI and **89**. As the concentration of **89** rose, the band intensity of β-tubulin increased, whereas that of EBI/β-tubulin adduct decreased. These results indicated that **89** and EBI competed for the colchicine site.

Lastly, molecular docking analysis was conducted to probe the interaction details of **89** with tubulin. Figure 4D visualized that **89** could fully occupy the colchicine site with high affinity (docking score: −8.682), and it formed broad hydrophobic interactions with 16 amino acid residues (Fig. 4E). In addition, three charged (one negative and two positive) and six polar interactions were also resolved. The docking results and detailed interactions of **89** with tubulin were provided in Fig. 4D, E and Supplementary Table S3.

### Compound 89 suppressed tumor cell proliferation via disrupting microtubular network and organization

**Fig. 5:**
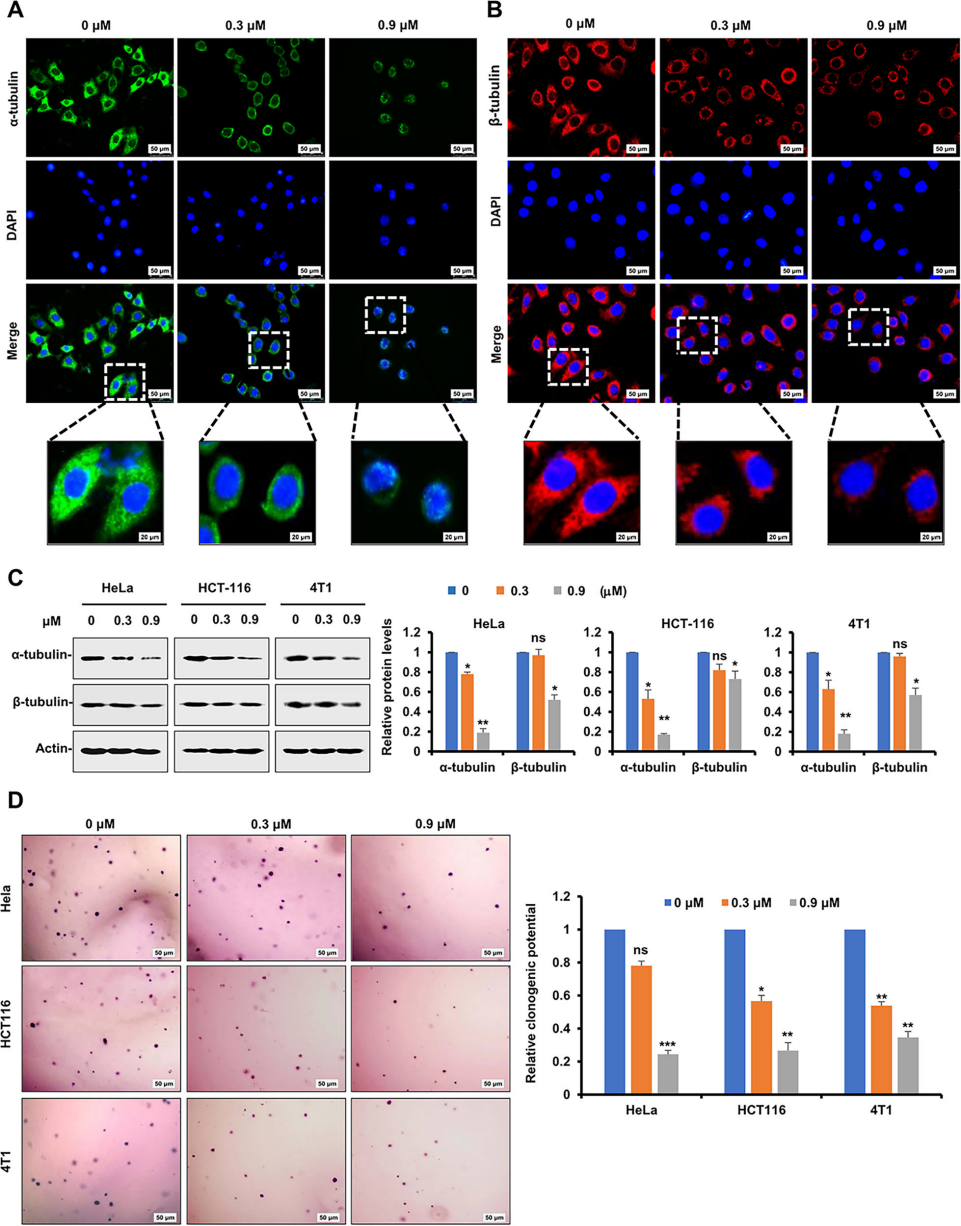
Compound 89 suppressed tumor cell proliferation via disrupting microtubule network and organization. **A**, **B** HeLa cells were treated with increasing concentrations of **89** for 24 h. Tubulin was stained with anti-α-tubulin (green) and anti-β-tubulin (red) antibodies, while the nucleus (blue) was stained with DAPI. The boxed areas were magnified to show clearer changes of the microtubule network in **89**-treated cells by immunofluorescence assay. **C** HeLa, HCT116 and 4T1 cells were treated with indicated concentrations of **89** and the expressions of α and β-tubulin were detected by Western immunoblotting assay with the indicated antibodies. Protein expressions were quantitated by densitometry and normalized against that of actin. **D** HCT116 cells were treated with increasing concentrations of **89**, and then soft agar colony formation assay was performed. Bars represent mean ± SD of three independent experiments. ns, no significant difference, *p < 0.05, **p < 0.01, ***p < 0.001 versus the control group.

As microtubules represent one of the major components of eukaryotic cytoskeleton, the dynamic balance of its assembly from α and β-tubulin is very important to maintain cell morphology and survival. Immunofluorescence staining experiment was then employed to inspect the effect of **89** on the microtubular network in living cells. The photographs in Fig. 5A, B clearly demonstrated that in the blank control groups, the microtubules were well-organized and spanned the entire cell to support the structure and shape of cells. In contrast, treatment with **89** resulted in disorganized microtubular networks and cellular shrinkage. Furthermore, immunoblotting experiment was conducted to assess the effect of **89** on α and β-tubulins, and the results in Fig. 5C revealed that the expressions of both proteins were notably downregulated upon treatment of **89**. Moreover, colony formation assay in soft agar was further conducted, and it was demonstrated by the experimental pictures (Fig. 5D) that the proliferation of tumor cells was significantly blocked by administration with **89**. Collectively, these results corroborated that **89** inhibited tumor cell proliferation by disrupting the network and organization of microtubules.

### Compound 89 regulated microtubular organization by inhibiting PI3K/Akt signaling

**Fig. 6:**
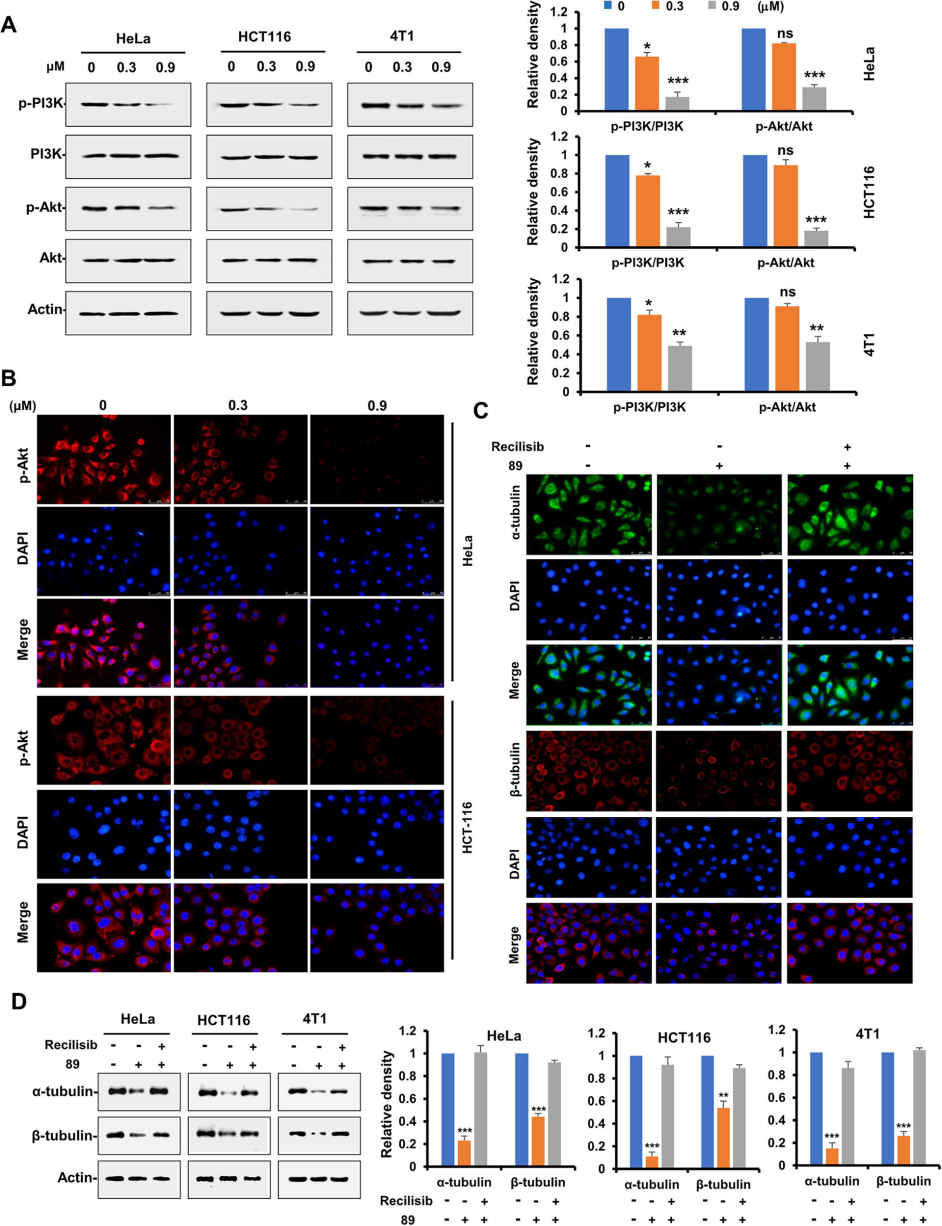
Compound 89 regulated microtubule stability via inhibiting PI3K/Akt signaling pathway. **A** HeLa, HCT116 and 4T1 cells were treated with indicated concentrations of **89**, and the expressions of key proteins of PI3K/Akt signaling were detected by Western immunoblotting assay with the indicated antibodies. **B** HeLa and HCT116 cells were treated with increasing concentrations of **89** for 24 h, and cells were stained for p-Akt (red) and the nucleus (blue) was stained with DAPI by immunofluorescence assay. **C** HeLa cells were pretreated with **89** (1.2 μM) for 12 h, then cells were treated with the PI3K activator recilisib (10 μM) for 12 h. Tubulin was stained with the anti-α-tubulin (green) and anti-β-tubulin (red) antibodies, while the nucleus (blue) was stained with DAPI by immunofluorescence assay. **D** HeLa cells were pretreated with **89** (1.2 μM) for 12 h, and then cells were treated recilisib (10 μM) for 12 h, and the expressions of α-tubulin and β-tubulin were detected by Western immunoblotting assay with the indicated antibodies.

As a classical signaling cascade involved in the migration and invasion of cancer cells, the PI3K/Akt pathway had been reported to participate in the stabilization of microtubules [30]. Therefore, whether the influence of **89** on microtubule is also related to the regulation of PI3K/Akt signaling was further inspected. Immunoblotting analysis was used to measure the total and phosphorylated levels of PI3K and Akt proteins in HeLa, HCT116, and 4T1 cells. The results showed significant reduction in the expressions of p-PI3K and p-Akt upon treatment of **89**, while the overall levels of PI3K and Akt remained unchanged (Fig. 6A). In addition, immunofluorescence experiment also confirmed the downregulation of **89** on the p-Akt expression in HeLa and HCT116 cells (Fig. 6B). Meanwhile, recilisib as an activator of PI3K signaling was thus applied to validate the influence of **89** on microtubules. As revealed by Fig. 6C from immunofluorescence analysis, the impaired microtubule network by **89** could be well recovered by further administration of recilisib in HeLa cells. In addition, the downregulated protein expression of α-tubulin and β-tubulin by **89** could also be reversed by treatment of recilisib according to Western blotting assay (Fig. 6D). Collectively, these results demonstrated that **89** could disturb the microtubule network by inhibiting PI3K/Akt pathway.

### In vivo antitumor and target verification studies of 89

**Fig. 7:**
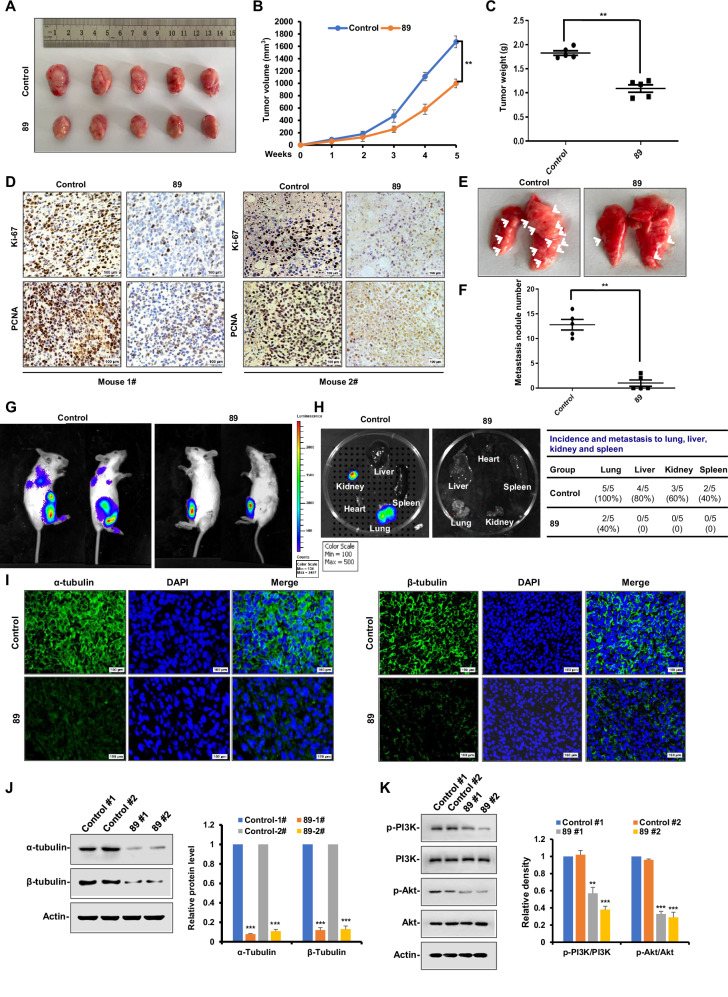
Compound 89 inhibited tumor growth and metastasis in vivo. **A** Representative images of the primary tumors removed from mice after administration of **89** (10 mg kg^−1^) or PBS once every 2 days for 28 day. **B** Primary tumor volume was measured each week (**p < 0.01). **C** Primary tumor weight in each group was measured (**p < 0.01). **D** Primary tumors were fixed and paraffin embedded. Five-micrometer (5 μm) sections were analyzed by IHC staining using anti-Ki-67 and anti-PCNA antibodies. Scale bar, 100 μm. **E**, **F** Metastatic lung nodules were visualized and then counted manually, and the differences were evaluated with Student *t* test (**p < 0.01). **G** Ex vivo bioluminescence images were obtained for selected mice in each group to check the effect of **89** against distant metastasis. **H** The metastasis incidence to distant organs was quantified. **I** Primary tumor sections were stained for α-tubulin (green) and β-tubulin (green), and nuclei were counterstained with DAPI (blue). Scale bar, 100 μm. **J** Primary tumors were lysed and applied to immunoblotting analysis using α-tubulin and β-tubulin antibodies, with actin as a loading control. **K** Primary tumors were lysed and applied to immunoblotting analysis using the indicated antibodies, with actin as a loading control. Bars represent mean ± SD of three independent experiments. *p < 0.05, **p < 0.01, ***p < 0.001 versus the control grou*p*.

Subsequently, we utilized the orthotopic autologous transplantation mouse model described earlier to conduct an in vivo anti-tumor evaluation on **89** [31], and the experimental animals were given intraperitoneal administration with 10 mg·kg^−1^ **89** or only PBS (control group) once every two days for 4 weeks. As shown in Fig. 7A–C, treatment of **89** markedly suppressed the tumor growth and reduced the tumor volume. According to the immunohistochemical results, the Ki-67 and PCNA levels in the primary tumor tissues were markedly reduced in the **89**-treated group, indicating the inhibition of **89** against the tumor cell proliferation in vivo (Fig. 7D). In addition, **89** also inhibited the metastasis of tumor from breast to lung (Fig. 7E, F), in agreement with the findings from the in vitro assay. Meanwhile, observation under an ex vivo imaging system (Fig. 7G) revealed that tumor cells grew aggressively and metastasized to remote organs in randomly selected mice of the control group but not in those of the **89**-treated group. Subsequently, the major organs of mice were excised and imaged to detect the tumor presence. The statistics in Fig. 7H revealed that only two mice in the **89-**treated group exhibited obvious metastasis to the lung, with no further metastases observed. In comparison, the tumor progression in the untreated group was notably more aggressive, with all five mice developing severe metastasis, as evident in their lungs (*n* = 5), livers (*n* = 4), kidneys (*n* = 3) and spleens (*n* = 2). In addition, the α-tubulin and β-tubulin in tumor tissue from **89**-treated mice were also examined by immunohistofluorescence staining (Fig. 7I), showing severely reduced and disturbed microtubule networks. Also, **89** suppressed the expression of α-tubulin and β-tubulin in the tumor according to Western blot analysis (Fig. 7J), supporting its direct target in vivo as microtubule. Lastly, the reduced levels of p-PI3K and p-Akt in the tumor tissues from **89**-treated group corroborated the downregulation of **89** on PI3K/Akt signaling (Fig. 7K), which suggested that the tumor growth and metastasis inhibitory effect of **89** also correlated with the suppression of this pathway in vivo.

### Compound 89 showed antitumor activity in human tumor organoids

**Fig. 8:**
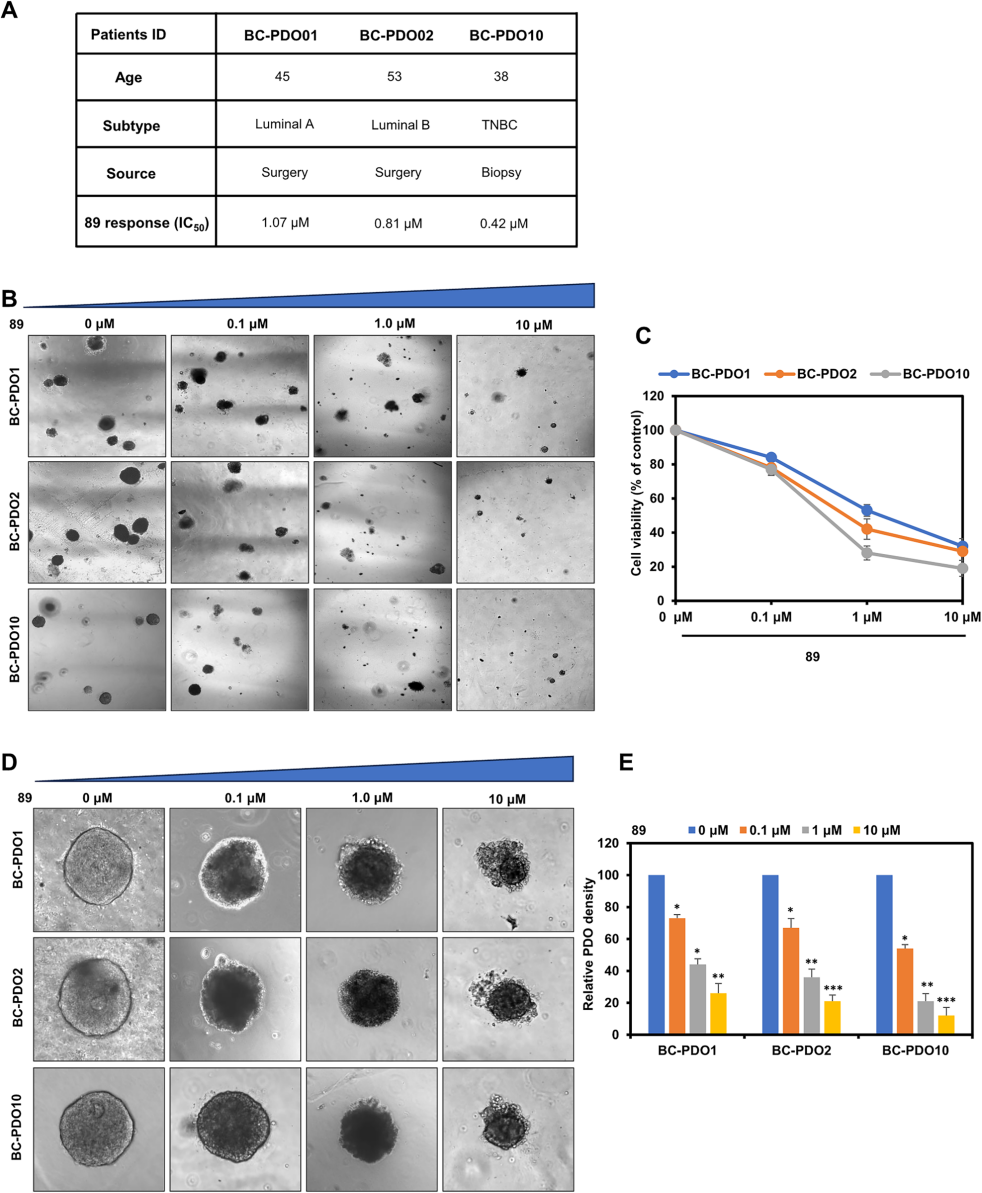
Compound 89 showed antitumor activity in human tumor organoids. **A** Patients’ basic information of the established PDOs and the responses to **89**. **B** The established PDOs were treated with increasing concentrations of **89** for 24 h, and representative images of the morphology of the PDOs were shown (scale bar, 20 μm). **C** The line graphs showed the cell viability of the PDOs treated with different concentrations of **89**. **D** Bright-field microscopy images showed the changes in the size and status of the PDOs after treatment with different concentrations of **89** (scale bar, 10 μm). **E** Relative PDOs density in response to different concentrations of **89**. *p < 0.05, **p < 0.01, ***p < 0.001.

The living cancer cells were separated from the patient breast tumor tissues via mechanical destruction followed by enzymatic digestion, and patient-derived organoids (PDOs) were successfully established, with the clinical details of the three patients (BC-PDO1, BC-PDO2, BC-PDO10) provided (Fig. 8A). The acquired PDOs were treated with varying doses of **89** and they exhibited different responses to this drug administration. The results in Fig. 8A, B revealed that the three PDOs were all sensitive to **89**, with IC_50_ values of 1.07 (BC-PDO1), 0.81 (BC-PDO2) and 0.42 (BC-PDO10) μM, respectively. Subsequent investigations showed that **89** could decrease the cell viability of the PDOs and the effect was positively correlated with the concentrations (Fig. 8C). Additionally, as the concentration of **89** increased, notable changes in the size and morphology of the PDOs were observed accordingly. As demonstrated in Fig. 8D, the cell clusters within the three organoids decreased in size, the organoids became smaller, the cell mass broke down into individual cells, and eventually, some cells fragmented. Treatment with **89** also reduced the overall density of the PDOs (Fig. 8E). These findings collectively indicated that compound **89** could act as a good lead molecule to be developed into more potential candidates for future antitumor studies.

### Compound 89 showed no systematic toxicity on mice

**Fig. 9:**
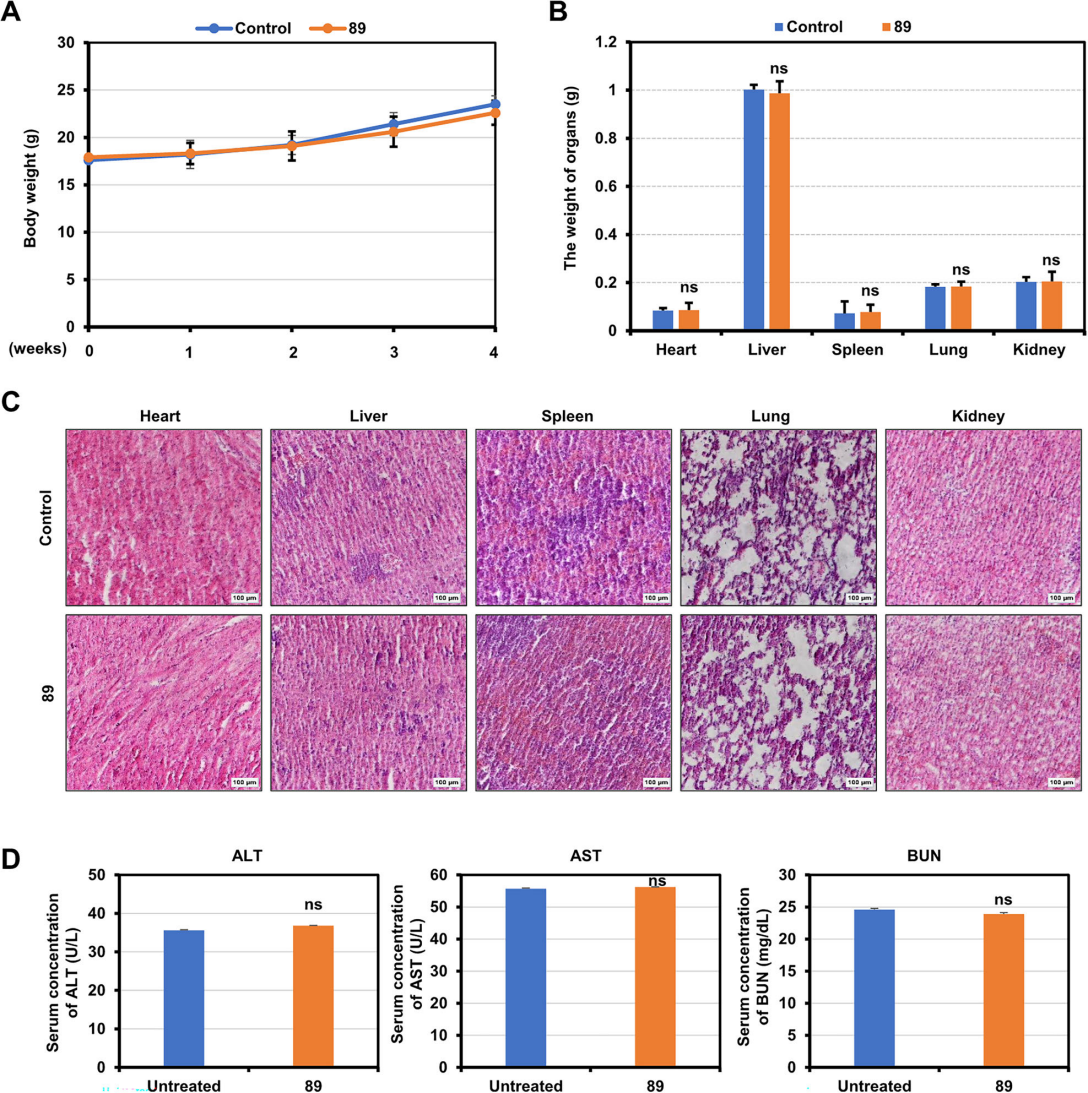
Compound 89 showed no systematic toxicity on mice. **A** Compound **89** or PBS (control) were administered to normal BALB/c mice at 10 mg kg^−1^ once every two days for 28 day, and the mice’s body weight was monitored once a week. Each treatment group consisted of five mice. **B** Major organs were removed and the weight was measured from the executed mice after the last drug treatment. **C** Major organs of randomly selected mice from different groups were stained with H&E (scale bar 100 μm) and observed under an inverted microscope (IX-71, Olympus, Tokyo, Japan). **D** Blood samples were collected 24 h after the last treatment and the serum concentrations of ALT, AST and BUN were measured using Fuji DRI-CHEM 7000i (Fujifilm, Japan).

To assess its possible toxicity on animals, **89** (10 mg·kg^−1^) or PBS was administrated every other day to healthy BALB/c mice for 28 consecutive days. The mice’s body weight was measured every seven days and organ weight measured at the completion of the experiment. Excitingly, there was no observed loss in the weight of both body and major organs of the animals treated with **89** (Fig. 9A, B). Additionally, histological analysis showed that, compared with the PBS group, compound **89** did not cause observable impairment to the main animal organs (liver, heart, lung, kidney and spleen, Fig. 9C). Furthermore, the serum levels of aspartate aminotransferase (AST), alanine aminotransferase (ALT, indicators of liver function) and blood urea nitrogen (BUN, indicator of both liver and kidney health) of the tested mice were all measured after the experiment. As illustrated in Fig. 9D, compound **89** showed no significant impact on the levels of the three indicators. These data collectively suggested that treatment of **89** did not induce notable toxicity in the experimental animals.

## Discussion

Over the past several decades, tubulin inhibitors, including microtubule-destabilizing and stabilizing molecules have been widely applied as chemotherapeutics for cancer prevention [24, 32, 33]. At present, U.S. FDA-approved tubulin inhibitors, including taxanes and vinca alkaloids, continue to be utilized in clinical settings to treat hematological malignancies and various solid tumors [34, 35]. However, the challenges of adverse effects and drug resistance persist due to prolonged usage. Owing to the intrinsic advantage of these traditional chemotherapies, such as their broad antitumor spectrum, continuing efforts have been put into the research and development of new type of tubulin inhibitors to circumvent the aforementioned deficiencies. In this study, we excavated from a huge compound library containing 200,340 molecules, a new potent tubulin aggregation inhibitor (**89**). This molecule significantly disrupted the structure and network of microtubules and displayed promising tumor growth and metastasis inhibitory effects both in vitro and in vivo.

Taxane, vinca, and colchicine binding sites are the three most extensively investigated target sites of microtubules [36]. Previous preclinical work suggested that the colchicine-site MTAs exerted promising therapeutic advantages compared with the taxane-site and vinca-site MTAs [37], and their much simpler structures greatly facilitated the syntheses and further structural modification. Therefore, studies on colchicine-site inhibitors have garnered significant attention in recent years [38, 39]. Currently, several candidate molecules that bind to the colchicine site, including ABT-751 and CA-4P, are undergoing clinical practice for cancer treatment [40, 41]. However, they have not been approved by the FDA because of side effects [19, 37, 42]. Therefore, the development of more colchicine-site MTAs with improved efficacy and reduced toxicity is still of great importance. In the present work, a virtual screening based on the taxane and colchicine sites for new MTAs identified **89**, a nicotinic acid derived synthetic compound, as a promising hit molecule. Further EBI competitive assay and molecular docking analysis demonstrated that **89** bound tightly to the colchicine site at the interface of two tubulin protein subunits.

As above discussed, known MTAs of the colchicine site, from the point of further drug development, incorporate obvious structural advantages compared with inhibitors of other binding sites, while excitingly compound **89** bears even better structural features than most previous colchicine-site inhibitors [38, 39]. Firstly, **89** does not possess so many electron donating groups (*e.g*., OH and OMe) like the former ones do, which brings it structural stability. Secondly, the nearly “bald” framework (including the pyridine, piperidine, piperazine and benzene rings) of **89** leaves enough space for further structural optimizations. Thirdly, the nicotinic acid moiety, as the core structure of **89**, widely exists as a natural compound especially in animals, and it may endow **89** with good biocompatibility and thus decent pharmacokinetic profile. Collectively, these structural properties make **89** an ideal lead molecule for further structural modification and optimization.

In conclusion, a novel tubulin inhibitor (**89**) has been identified from a commercially available compound library *via* virtual screening in the present study. Experimental investigations demonstrated that compound **89** efficiently suppressed the growth, blocked the migration, and induced the apoptosis and G_2_/M phase arrest of different tumor cells. In addition, it also suppressed the tumor growth and metastasis in vivo in mice and exerted promising antitumor capability in PDOs. Mechanistical studies validated that compound **89** functioned as a colchicine-site tubulin polymerization inhibitor and disrupted the dynamic equilibrium of tubulin assembly by inhibiting the PI3K/Akt pathway. To summarize, the present research has identified a novel template molecule that can be utilized in the development of next-generation MTAs for cancer treatment.

## Material and methods

### Source of compounds

The compounds used in this study were obtained from a commercially available Specs library (Zoetermeer, The Netherlands).

### Virtual screening for tubulin inhibitors

Molecular docking-based virtual screening was conducted using the Glide 5.5 program in the Maestro software package (Schrödinger LLC, New York, NY, 2015). Protein-ligand complexes of tubulin (PDB IDs: 4O2B and 5LXT) were prepared through the Protein Preparation Wizard panel. This preparation included hydrogen addition, charge assignment, and removal of water molecules not within proximity to the binding pocket, followed by restrained energy optimization. Binding sites were defined within a 15 Å radius surrounding the ligands (colchicine and discodermolide), and grid files were generated. Subsequently, compounds from the Specs library were docked into the defined grids using extra precision mode. The docking results were subjected to clustering analysis using Pipeline Pilot 7.5 software.

### Molecular docking of compound 89

Molecular docking analysis for compound **89** at the colchicine binding site followed the same procedures as described in the virtual screening protocol.

### Cell lines and reagents

Details regarding the cell lines and reagents are provided in the supplementary materials.

### Animal studies

BALB/c mice (6–8 weeks old) were acquired from the Institute of Laboratory Animal Science of the Chinese Academy of Medical Sciences (Beijing, China). All animal experiments adhered to the guidelines approved by Qingdao University’s Institutional Animal Care and Ethics Committee (QDU-AEC-2024075). For therapeutic evaluations, mice were divided into two groups (*n* = 5 per group). The experimental group received intraperitoneal injections of Compound **89** (10 mg·kg^−^^1^) every 2 days, while the control group was injected with PBS. Body weights were monitored weekly, and major organs were harvested for histological examination on day 28. An additional toxicity assessment was conducted on healthy mice under identical conditions.

### Cell viability assay

Cell viability was evaluated using the MTS assay [43]. HeLa, HCT116, and 4T1 cells were seeded in 96-well plates at a density of 5 × 10³ cells/well. After 24 h, cells were treated with varying concentrations of test compounds. Absorbance at 490 nm was recorded after 24 h. Each experiment was repeated three times in triplicate.

### Colony formation assay

HeLa, HCT116, and 4T1 cells were seeded in 6-well plates and treated with different concentrations of compound **89** after 12 h. Colonies were allowed to form over 1–2 weeks, with media refreshed every other day. Cells were fixed with 4% paraformaldehyde, stained with 0.1% crystal violet, and manually counted.

### Soft agar colony formation assay

Soft agar assay involved the preparation of 1.2% and 0.7% agar, mixed with equal volumes of cell culture medium supplemented with FBS and antibiotics. A 1.2% agar layer was solidified in 6-well plates, then treated tumor cells were mixed into the 0.7% agar layer and overlaid. Cells were cultured for 14 days, supplemented with medium every 3 days, stained with crystal violet, and visualized under a microscope (Olympus BX53, Tokyo, Japan).

### Cell cycle analysis

HeLa and HCT116 cells were treated with compound **89**, fixed in 70% ethanol, and stored at 4 °C for 24 h. Cells were stained with RNase and propidium iodide (PI) solution, incubated at 37 °C for 30 min, and analyzed using flow cytometry.

### Apoptosis analysis

Apoptosis was assessed via Annexin V-FITC/PI staining. HeLa, HCT116, and 4T1 cells were treated with increasing concentrations of compound **89**, stained using an apoptosis detection kit, and analyzed using flow cytometry.

### Wound healing assay

A scratch was created on confluent monolayers of HeLa, HCT116, and 4T1 cells. Cells were treated with compound **89** in serum-free medium, and migration was evaluated after 24 h by imaging the wound closure.

### Cell invasion assay

Invasion assays were performed using Matrigel-coated transwell chambers. Serum-starved HeLa, HCT116, and 4T1 cells were placed in the upper chamber, and compound **89** was added to both compartments. After 12 h, invaded cells were stained and counted.

### Western blotting

Western blotting assay was performed as described previously [44]. Briefly, cell lysates were prepared in RIPA buffer, and protein concentrations were measured using a BCA assay. Proteins were resolved via SDS-PAGE, transferred to PVDF membranes, blocked with BSA, and probed with specific primary and secondary antibodies. Signals were visualized using ECL reagents.

### Cellular thermal shift assay (CETSA)

Interaction between compound **89** and β-tubulin in living cells was analyzed by CETSA. HeLa cell lysates were treated with **89**, subjected to thermal denaturation, centrifuged, and the supernatant analyzed by Western blot.

### In vitro tubulin polymerization assay

The in vitro tubulin polymerization assay was performed according to the manufacturer’s instructions (Cytoskeleton, Cat# BK006P). Briefly, a series of concentrations of **89** (6.25, 12.5, 25, 50 and 100 μM), 10 μM paclitaxel, 10 μM colchicine and control (DMSO) were incubated with tubulin protein in 100 μL reaction buffer (pH 6.9) containing 80 mM piperazine-1,4-bisethanesulfonic acid, 2.0 mM MgCl_2_, 0.5 mM ethylene glycol tetraacetic acid, 15% glycerol, 1 mM guanosine-5′-triphosphate. The mixture was incubated at 37 °C in a microplate reader (Tecan Spark 10 M, Tecan, Austria) and the absorbance at 340 nm was recorded every 60 s for 1 h. Colchicine and paclitaxel were used as reference compounds.

### N,N’-Ethylenebis (EBI) competition assay

6-well plates were seeded with HeLa or HCT116 cells (2 × 10^5^) for 24 h, then the tumor cells were incubated with compound **89**, colchicine or DMSO for 2 h and afterward treated with EBI (100 μM) for 1.5 h. Finally, the cells were harvested and lysed, and the cell extracts were used for Western blotting analysis.

### Immunofluorescence staining

Cells were fixed, stained with primary and fluorescent secondary antibodies, and visualized using a fluorescence microscope.

### Histological and immunohistochemical (IHC) analysis

Histological and immunohistochemical analysis were performed as described previously [45]. In brief, major organs and tumor tissues were fixed, paraffin-embedded, and sectioned for H&E and IHC staining using antibodies against PCNA and Ki-67.

### Patient-derived organoid culture

Patients were informed before the surgery and agreed by written consent to donate tissues. All experiments were performed in accordance with the IRB committee’s regulations of Qingdao University on human subject research. All procedures were performed at the Affiliated Hospital of Qingdao University (QDU-HEC-2024044). The tissue was digested in 1 mL Dispase (Corning, diluted 1:5 in HBSS) and 1 mL Trypsin-EDTA (Invitrogen, 0.25%), followed by mechanical dissociation and passing through a 100 μm cell strainer (Falcon). Then the cells were resuspended in 40 μL Cultrex Reduced Growth Factor Basement Membrane Extract (BME), Type 2 (R&D Systems, Cat# 3533–010-02) and seeded into Matrigel in a well of a pre-warmed 24-well flat-bottom cell culture plate (Corning). Then, the cells were incubated for 20 min in a cell incubator (37 °C with 5% CO_2_) to solidify the Matrigel and were subsequently overlaid with 400 μL of complete human organ culture medium. Medium was changed every 4 days and organoids were passaged every 1–4 weeks. All organoid lines were tested negative by the MycoAlert mycoplasma detection kit (Cat# LT07-318, Lonza). PDOs were observed and photographed as required.

### Statistical analysis

All experiments except the in vivo study were structured with control and experimental groups and repeated at least three times. Data were presented as mean ± SD of at least three independent experiments. The “*n*” represents the number of biological replicates as indicated in the figure legends. A Student’s *t* test was used to compare the data between two groups (*p* < 0.05 was considered to show statistical significance) unless otherwise indicated.

## Supplementary information

Supplementary information

Full and uncropped western blots

## Data Availability

Data will be made available on request.
