# Supplementary material for: Discovery of a novel potent tubulin inhibitor through virtual screening and target validation for cancer chemotherapy
Source: Cell Death Discov. 2025 Aug 19;11:392. doi: 10.1038/s41420-025-02679-3 (PMC12365163; doi:10.1038/s41420-025-02679-3)
Supplement: Supplementary file 1 — Supplementary information [file 41420_2025_2679_MOESM1_ESM.docx]

**Discovery of a Novel Potent Tubulin Inhibitor through Virtual Screening and Target Validation for Cancer Chemotherapy**

Peipei Shan^1,#,^ ^✉^, Kai-Lu Liu^2,#^, Xiu Jiang^3^, Guangzhao Zhou^4^, Kongkai Zhu^2,5,^ ^✉^, and Hua Zhang ^1,2,^ ^✉^

^1^ Institute of Translational Medicine, the Affiliated Hospital of Qingdao University, College of Medicine, Qingdao University, Qingdao 266021, China

^2^ School of Biological Science and Technology, University of Jinan, Jinan 250022, China

^3^ Qingdao Central Hospital, University of Health and Rehabilitation Sciences, Qingdao 266042, China

^4^ Department of Anesthesiology, The Affiliated Hospital of Qingdao University, Qingdao 266002, China

^5^ Advanced Medical Research Institute, Cheeloo College of Medicine, Shandong University, Jinan 250012, China

^#^ These authors contributed equally to this work.

^✉^ Corresponding authors, E-mail addresses: [shanpeipei@qdu.edu.cn](mailto:shanpeipei@qdu.edu.cn) (P. Shan), [hkhhh.k@163.com](mailto:hkhhh.k@163.com) (K. Zhu), bio_zhangh@ujn.edu.cn (H. Zhang)

KEYWORDS: nicotinamide analogue; tubulin inhibitor; virtual screening; antitumor chemotherapy; PI3K/Akt

**Table of Contents**

**Experimental**………………………………………………………………………….3

**Cell lines and reagents**………………………………………………………………..3

**Table S1.** The Specs codes of 93 candidate compounds from virtual screening………4

**Table S2.** Inhibitory rates (%) of 17 analogues of **89** against Hela and HCT116 cells..5

**Table S3.** Docking results and detailed interactions of **89** with tubulin……………….7

Figure S1. Cytotoxic effects of compound 89 against a panel of tumor cell lines other than the three mentioned ones in the main text ……………….………………………7

**Figure S2**. Compound **89** induced G2/M phase arrest in 4T1 cell lines……………….7

**Materials and methods**

**Cell lines and reagents.** HeLa, HCT116 and 4T1 cells were obtained from the American Type Culture Collection (Manassas, VA, USA). HeLa and HCT116 cells were cultured in DMEM supplemented with 10% (v/v) fetal bovine serum (FBS), 100 U/mL penicillin, 100 μg/mL streptomycin, and maintained at 37 °C in a humid environment with 5% CO_2_. 4T1 cells were maintained in RPMI 1640 (Cat# MA0214, Meilunbio) medium with 10% (v/v) fetal bovine serum (Cat# PWL0001, Meilunbio), and maintained at 37 °C in a humid environment with 5% CO_2_. All cell lines tested negative for Mycoplasma contamination (Mycoplasma Detection Kit, TransGen Biotech). The cells were passaged for fewer than 6 months after resuscitation and were authenticated prior to use by short tandem repeat profiling. Matrigel was purchased from BD Bioscience (Pasadena, CA, USA). Antibody against PCNA (Cat# Ab19166) for WB and IHC assays was purchased from Abcam. Antibody against actin (Cat# A5316) for WB assay was purchased from Sigma. Antibodies against Cdc25c (Cat# 4688), Cyclin B1 (Cat# 12231), Bcl-2 (Cat# 3498), Bcl-XL (Cat# 2792), cleaved PARP (Cat# 9544P), E-cadherin (Cat# 4065), Vimentin (Cat# 5741), ZEB1 (Cat# 3396), α-tubulin (Cat# 2144), β-tubulin (Cat# 2146), Akt (Cat# 9272), p-PI3K (Cat# 17366) and PI3K (Cat# 4257) were purchased from Cell Signaling Technology Inc. Antibody against CDK1 (Cat# ab133327) for WB assay was purchased from Abcam. Antibody against p-Akt (Cat# 9271) for WB and IF assays was purchased from Cell Signaling Technology Inc. Antibody against Ki-67 (Cat# 9129) for IHC assay was purchased from Cell Signaling Technology Inc. Recilisib was purchased from MedChemExpress (MCE, Cat# 01210). All tested compounds were dissolved in DMSO and stored at −20 °C as small aliquots.

**Table S1.** The Specs codes of 93 candidate compounds from virtual screening.

| Testing code | Specs code | Testing code | Specs code | Testing code | Specs code |
| --- | --- | --- | --- | --- | --- |
| **1** | AG-205/32458033 | **32** | AP-970/12269318 | **63** | AG-690/15436701 |
| **2** | AO-080/43441570 | **33** | AG-227/41299482 | **64** | AB-323/13887317 |
| **3** | AJ-292/40706485 | **34** | AG-690/12243059 | **65** | AR-434/43295101 |
| **4** | AB-323/13887082 | **35** | AQ-149/13766835 | **66** | AP-970/43375087 |
| **5** | AS-871/43477963 | **36** | AK-778/43206412 | **67** | AG-690/12411002 |
| **6** | AS-662/43471423 | **37** | AG-205/41005535 | **68** | AK-968/41926298 |
| **7** | AO-476/43415679 | **38** | AO-080/43441583 | **69** | AO-080/43441899 |
| **8** | AI-204/31689056 | **39** | AE-848/30897050 | **70** | AT-417/43484995 |
| **9** | AT-417/43485024 | **40** | AP-970/43492226 | **71** | AG-401/43287209 |
| **10** | AK-968/14001102 | **41** | AP-853/42160592 | **72** | AQ-086/41227846 |
| **11** | AG-690/40720509 | **42** | AO-365/43474735 | **73** | AN-646/40633592 |
| **12** | AK-968/14004590 | **43** | AE-848/34304040 | **74** | AO-799/43505399 |
| **13** | AO-080/43441910 | **44** | AG-667/11912014 | **75** | AO-476/43417706 |
| **14** | AP-013/40848470 | **45** | AO-365/43474412 | **76** | AQ-750/41790370 |
| **15** | AG-205/12125146 | **46** | AT-051/43422643 | **77** | AK-918/42813919 |
| **16** | AO-476/43421060 | **47** | AK-778/43420919 | **78** | AE-848/12529001 |
| **17** | AI-204/31728036 | **48** | AO-365/43472833 | **79** | AK-968/40708181 |
| **18** | AG-205/37106180 | **49** | AN-329/43449390 | **80** | AN-465/43013178 |
| **19** | AG-690/33073020 | **50** | AE-848/32589053 | **81** | AN-329/43449087 |
| **20** | AF-399/40684382 | **51** | AP-853/42000730 | **82** | AE-848/12918190 |
| **21** | AP-263/43411808 | **52** | AJ-333/25006173 | **83** | AO-365/40105693 |
| **22** | AM-807/12426030 | **53** | AM-807/14145004 | **84** | AS-871/41500791 |
| **23** | AO-080/40818743 | **54** | AP-970/43482206 | **85** | AO-476/43362745 |
| **24** | AG-401/42008201 | **55** | AN-329/43448270 | **86** | AR-685/43362930 |
| **25** | AP-970/41681030 | **56** | AF-399/15539271 | **87** | AP-970/41851538 |
| **26** | AK-080/43416907 | **57** | AP-970/41401637 | **88** | AR-422/41979229 |
| **27** | AO-476/14976005 | **58** | AP-970/43482168 | **89** | AI-204/31693056 |
| **28** | AT-662/43485225 | **59** | AG-690/15438077 | **90** | AO-476/43417572 |
| **29** | AJ-292/41686549 | **60** | AO-080/43441697 | **91** | AO-476/41339622 |
| **30** | AO-080/43441647 | **61** | AP-970/43492094 | **92** | AO-080/43441492 |
| **31** | AK-968/37077044 | **62** | AP-263/43370992 | **93** | AM-760/13743006 |

**Table S2.** Inhibitory rates (%) of 17 analogues of **89** against Hela and HCT116 cells.

| Specs codes | testing codes | structures | Hela | HCT116 |
| --- | --- | --- | --- | --- |
| AI-204/31693057 | **A1** |  | 9.9 (0.3 μM)  16.5 (0.9 μM) | 8.6 (0.3 μM)  17.2 (0.9 μM) |
| AI-204/43372073 | **A2** |  | 7.1 (0.3 μM)  17.7 (0.9 μM) | 1.4 (0.3 μM)  5.3 (0.9 μM) |
| AI-204/31693054 | **A3** |  | -15.1 (0.3 μM)  -34.5 (0.9 μM) | -17.7(0.3 μM)  -24.8(0.9 μM) |
| AI-204/31693055 | **A4** |  | 3.5 (0.3 μM)  14.7 (0.9 μM) | 18.8 (0.3 μM)  22.4 (0.9 μM) |
| AI-204/43489623 | **A5** |  | 0.64 (0.3 μM)  43.7 (0.9 μM) | 3.1 (0.3 μM)  37.6 (0.9 μM) |
| AJ-333/36117058 | **A6** |  | 2.4 (0.3 μM)  52.9 (0.9 μM) | 9.8 (0.3 μM)  31.1 (0.9 μM) |
| AJ-333/36117059 | **A7** |  | -8.6 (0.3 μM)  -1.9 (0.9 μM) | -4.2 (0.3 μM)  -8.7 (0.9 μM) |
| AG-670/12607016 | **A8** |  | 15.4 (0.3 μM)  11.5 (0.9 μM) | 1.4 (0.3 μM)  3.0 (0.9 μM) |
| AH-262/15227007 | **B1** |  | 3.1 (0.3 μM)  8.9 (0.9 μM) | 3.6 (0.3 μM)  6.0 (0.9 μM) |
| AI-204/31693035 | **B2** |  | 8.8 (0.3 μM)  11.3 (0.9 μM) | 2.3 (0.3 μM)  5.6 (0.9 μM) |
| AJ-333/09216048 | **B3** |  | 11.8 (0.3 μM)  51.2 (0.9 μM) | 7.8 (0.3 μM)  32.7 (0.9 μM) |
| AK-968/12163777 | **B4** |  | -0.69 (0.3 μM)  -5.2 (0.9 μM) | -0.7 (0.3 μM)  -4.7 (0.9 μM) |
| AJ-333/09218012 | **B5** |  | 18.3 (0.3 μM)  18.7 (0.9 μM) | 19.0 (0.3 μM)  20.0 (0.9 μM) |
| AJ-333/36116044 | **B6** |  | 8.7 (0.3 μM)  26.6 (0.9 μM) | 2.9 (0.3 μM)  9.1 (0.9 μM) |
| AJ-333/36116045 | **B7** |  | 3.4 (0.3 μM)  -1.2 (0.9 μM) | 6.5 (0.3 μM)  7.3 (0.9 μM) |
| AK-968/12861071 | **B8** |  | 2.9 (0.3 μM)  15.7 (0.9 μM) | 6.7 (0.3 μM)  10.4 (0.9 μM) |
| AJ-333/36116049 | **C1** |  | 7.7 (0.3 μM)  0.6 (0.9 μM) | -1.9 (0.3 μM)  -3.9 (0.9 μM) |

**Table S3.** Docking results and detailed interactions of **89** with tubulin.

| **Docking score** | **Glide ligand efficiency** | **Hydrophobic interactions** | **Polar interactions** | **Charged interactions (negative)** | **Charged interactions (positive)** |
| --- | --- | --- | --- | --- | --- |
| −8.682 | −0.333 | 16 | 6 | 1 | 2 |

**Figure S1**. Cytotoxic effects of compound **89** against a panel of tumor cell lines other than the three mentioned ones in the main text.


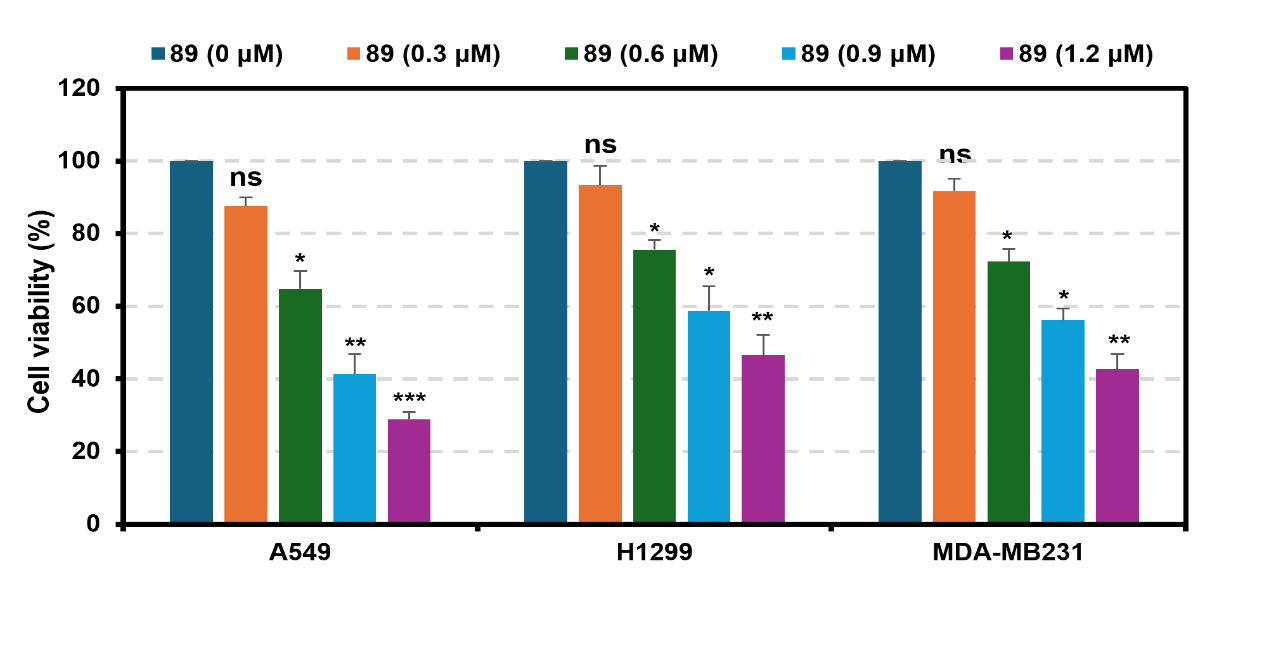


Figure legend. The tumor cells were treated with different concentrations of compound **89,** and MTS assay was performed after 24 h. The bars indicate mean ± SD (n = 3).

**Figure S2.** Compound **89** induced G2/M phase arrest in 4T1 cell lines.


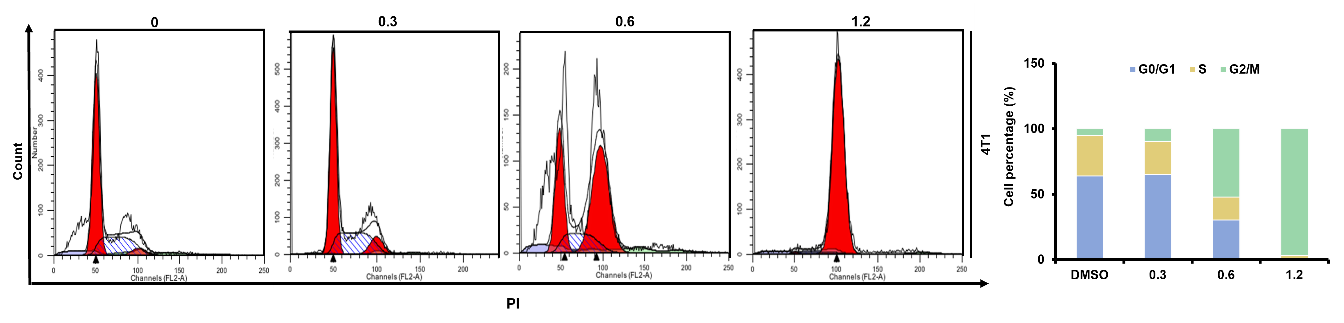


Figure legend. 4T1 cells were treated with indicated concentrations of **89** and co-incubated for 24 h. Cell population distribution was determined following PI staining and further analyzed by ﬂow cytometry. Bars represent mean ± SD of three independent experiments.
