# Supplementary material for: Discovery of a novel potent tubulin inhibitor through virtual screening and target validation for cancer chemotherapy
Source: Cell Death Discov. 2025 Aug 19;11:392. doi: 10.1038/s41420-025-02679-3 (PMC12365163; doi:10.1038/s41420-025-02679-3)
Supplement: Supplementary file 2 — Full and uncropped western blots [file 41420_2025_2679_MOESM2_ESM.pdf]

Figure 2B

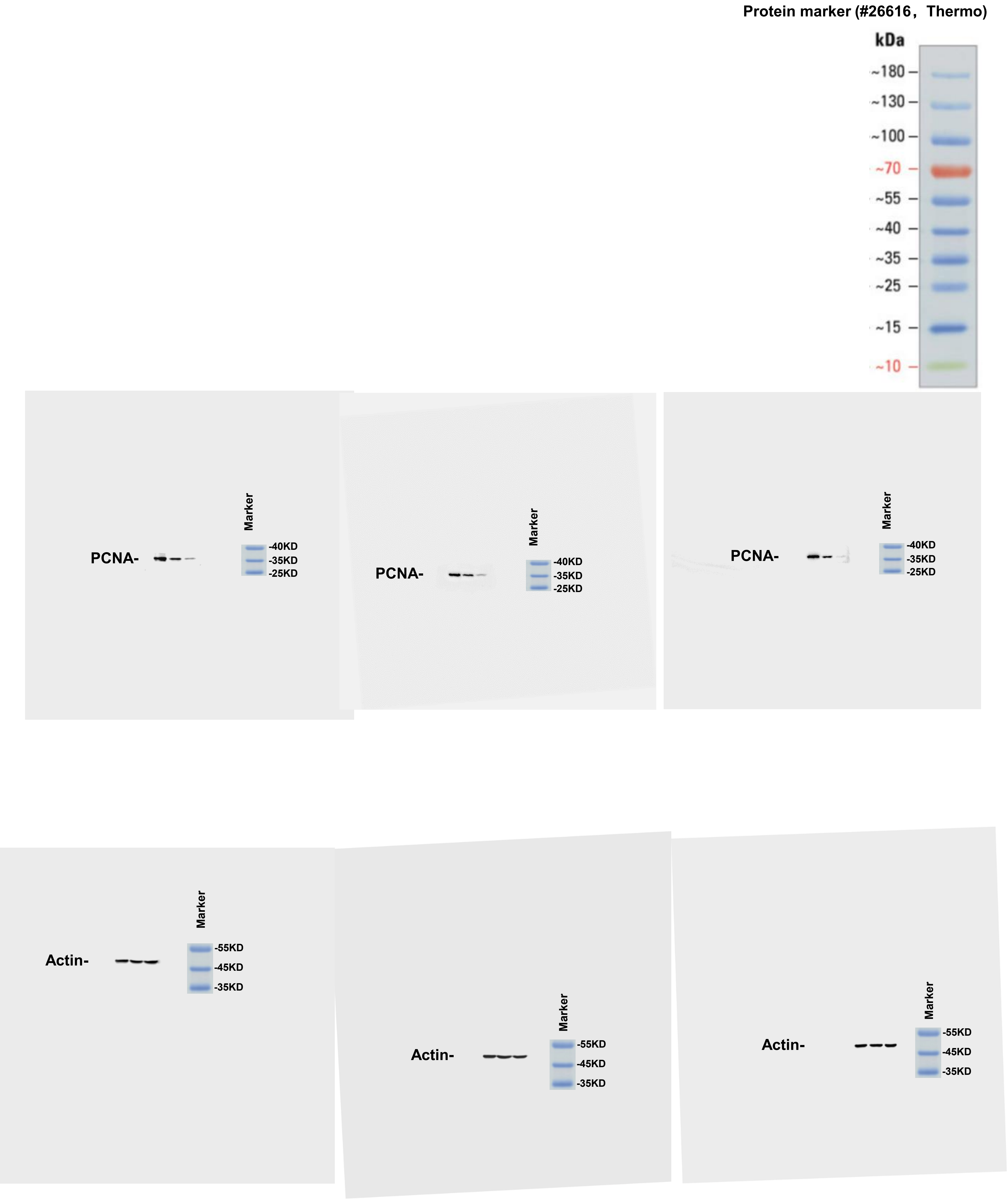

Protein marker (#26616，Thermo)
kDa
~180
~130
~100
~70
~55
~40
~35
~25
~15
~10
PCNA-
Marker
-40KD
-35KD
-25KD
PCNA-
Marker
-40KD
-35KD
-25KD
PCNA-
Marker
-40KD
-35KD
-25KD
Actin-
Marker
-55KD
-45KD
-35KD
Actin-
Marker
-55KD
-45KD
-35KD
Actin-
Marker
-55KD
-45KD
-35KD

Figure 2F

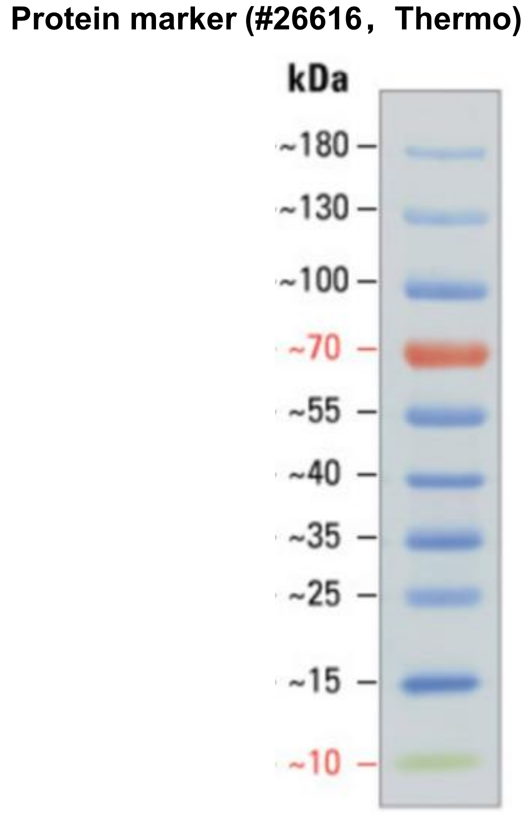
Protein marker (#26616，Thermo)
kDa
~180
~130
~100
~70
~55
~40
~35
~25
~15
~10

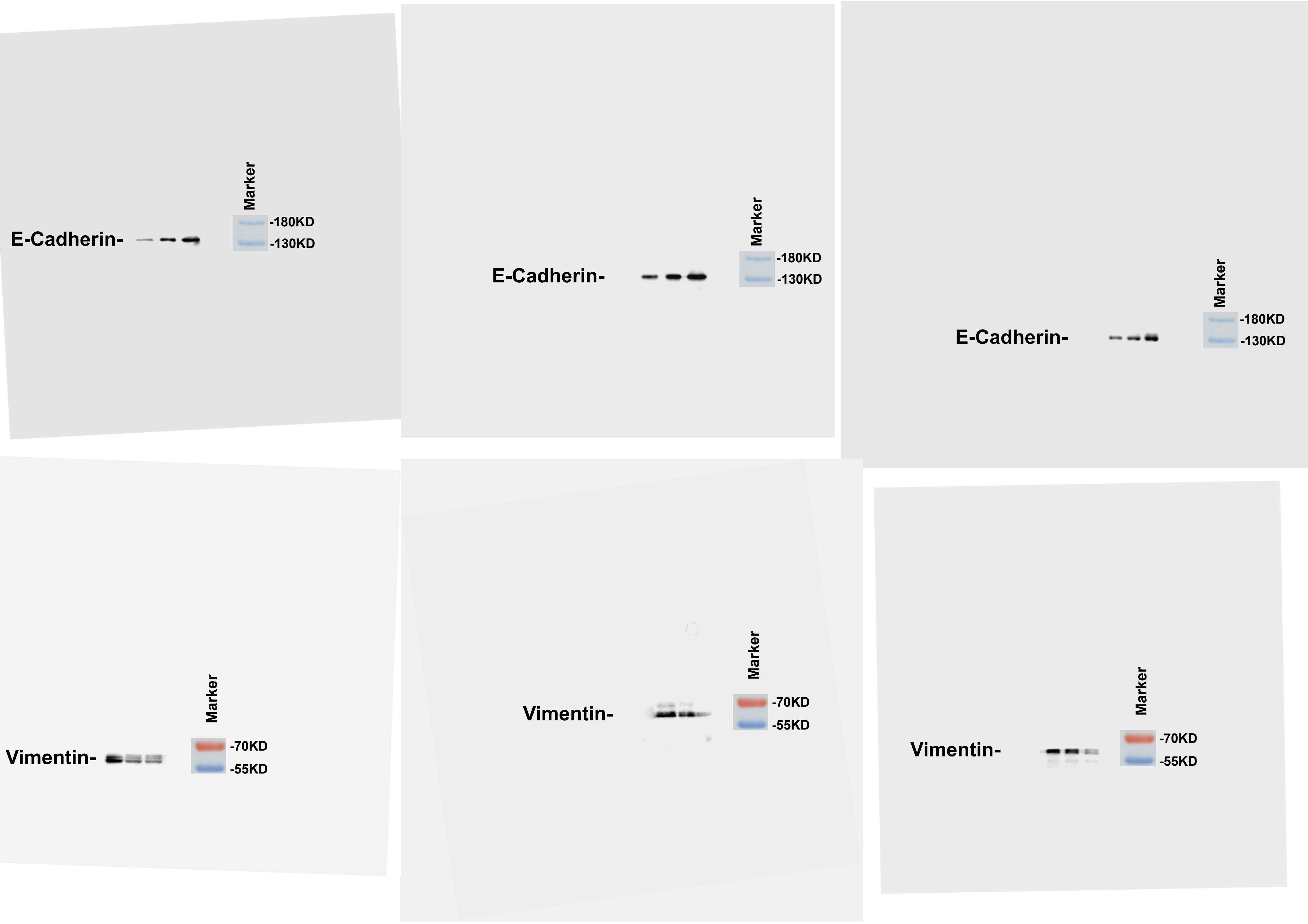
E-Cadherin-
Marker
-180KD
-130KD
E-Cadherin-
Marker
-180KD
-130KD
E-Cadherin-
Marker
-180KD
-130KD
Vimentin-
Marker
-70KD
-55KD
Vimentin-
Marker
-70KD
-55KD
Vimentin-
Marker
-70KD
-55KD

Figure 2F

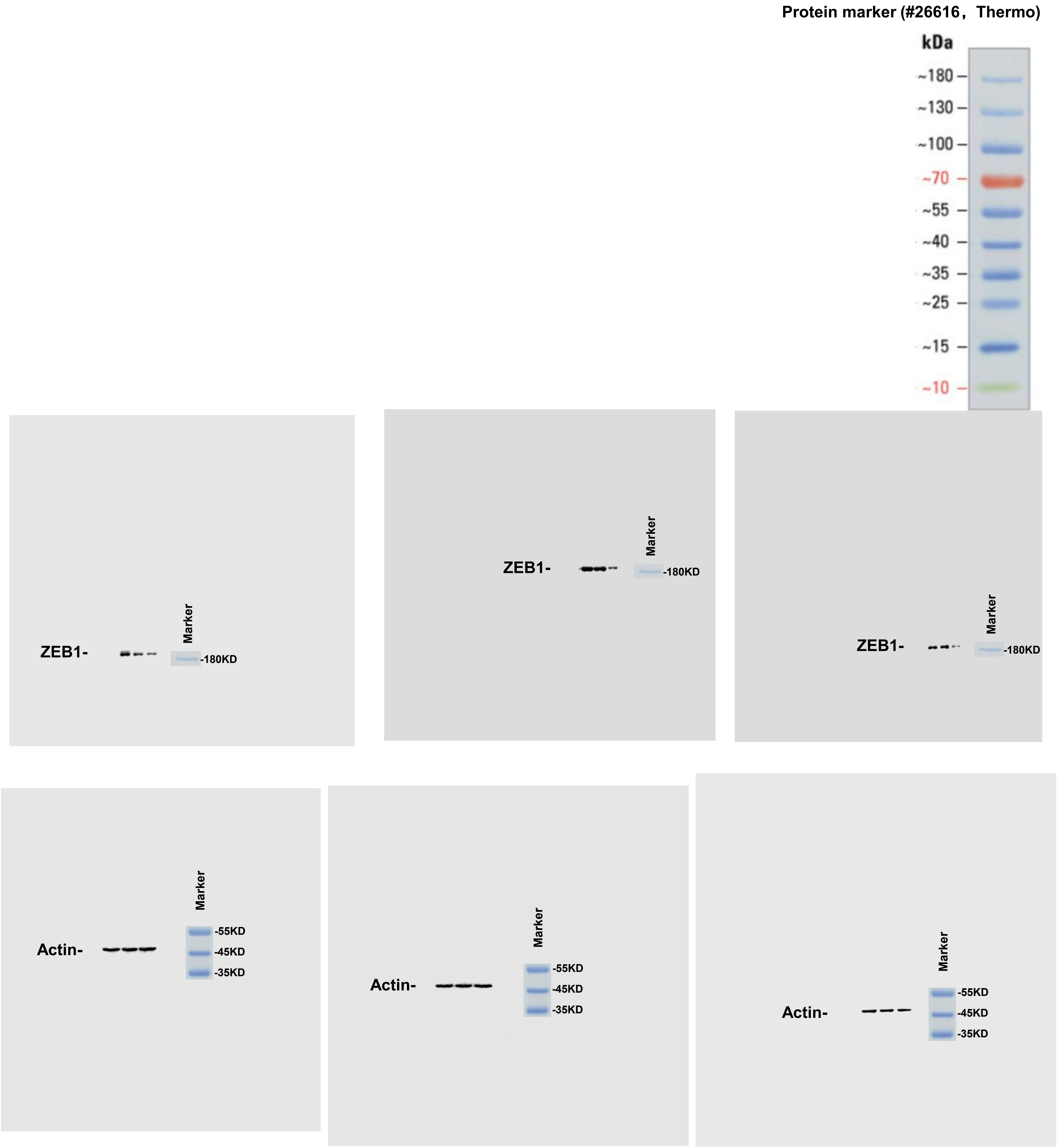

Protein marker (#26616，Thermo)
kDa
~180
~130
~100
~70
~55
~40
~35
~25
~15
~10
ZEB1-
Marker
-180KD
ZEB1-
Marker
-180KD
ZEB1-
Marker
-180KD
Actin-
Marker
-55KD
-45KD
-35KD
Actin-
Marker
-55KD
-45KD
-35KD
Actin-
Marker
-55KD
-45KD
-35KD

Figure 3B

Protein marker (#26616，Thermo)

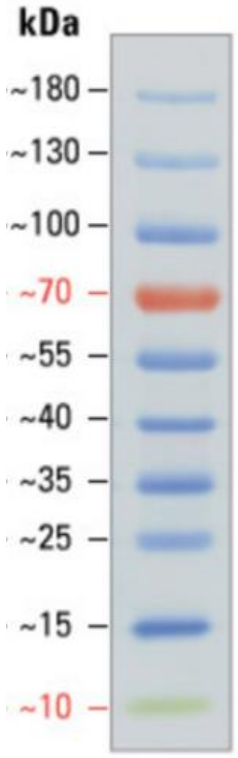

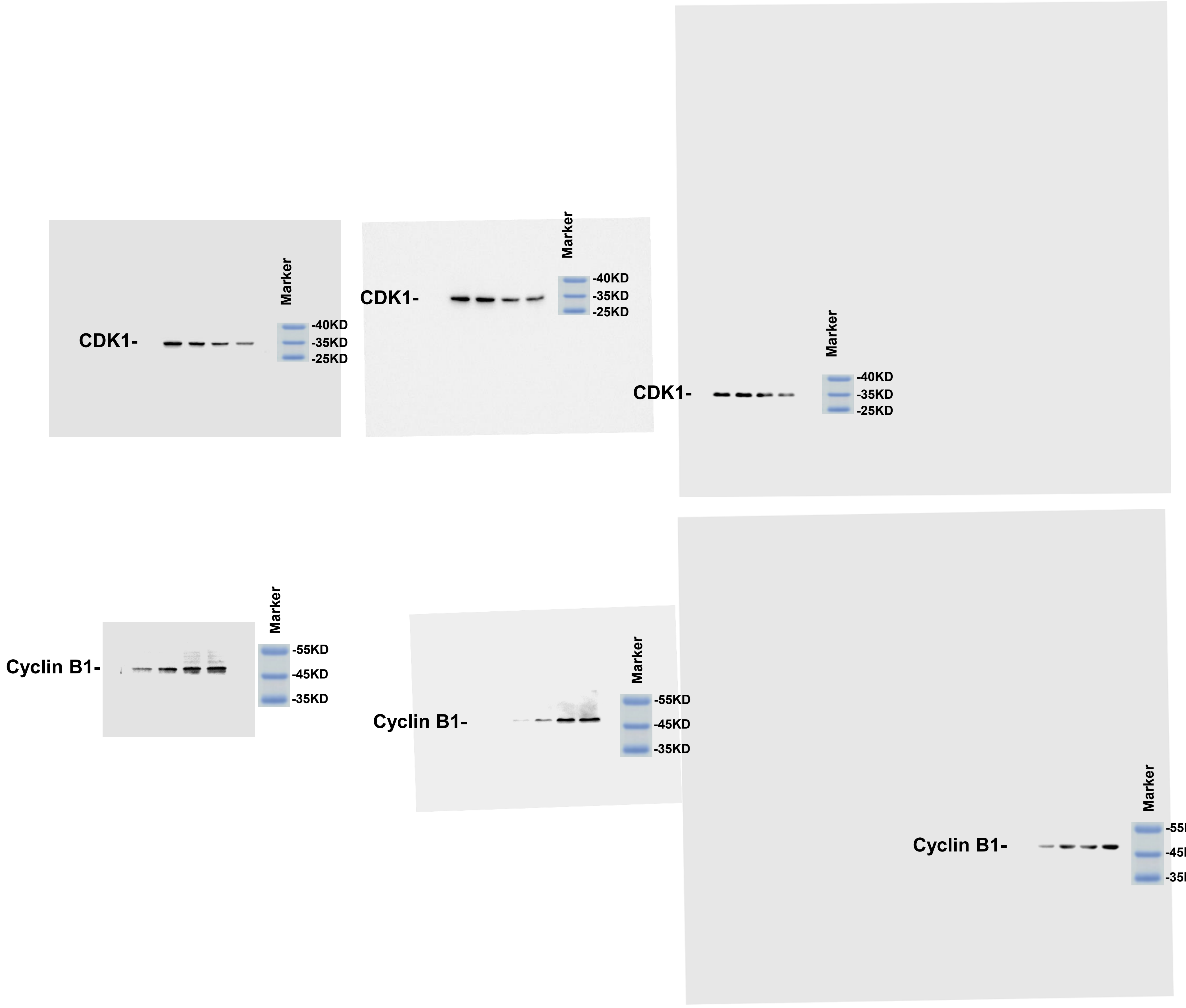

Figure 3B

Protein marker (#26616，Thermo)

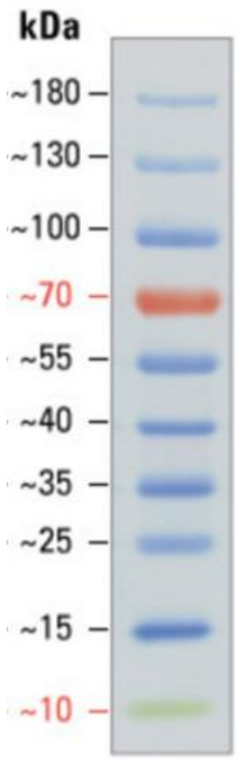

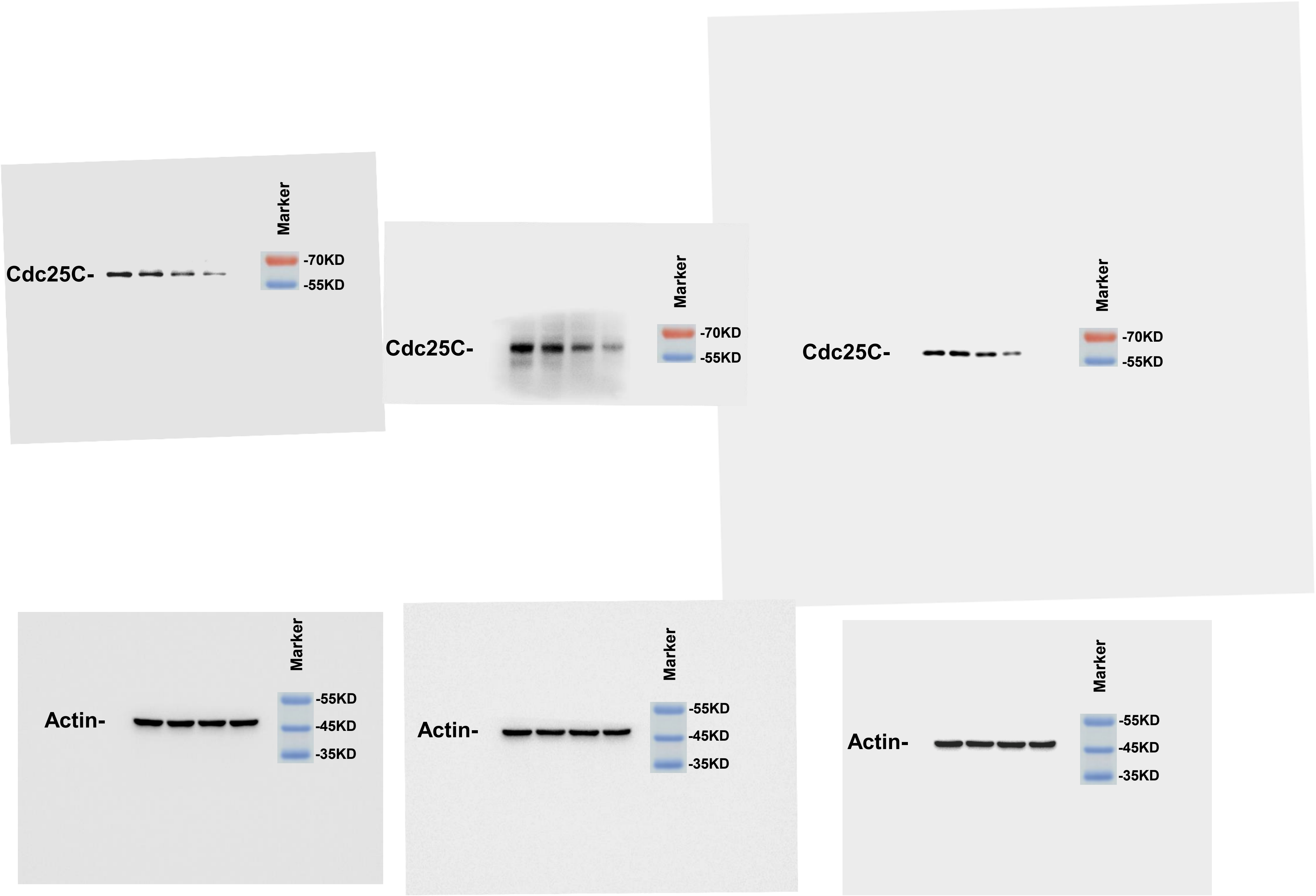

Figure 3E

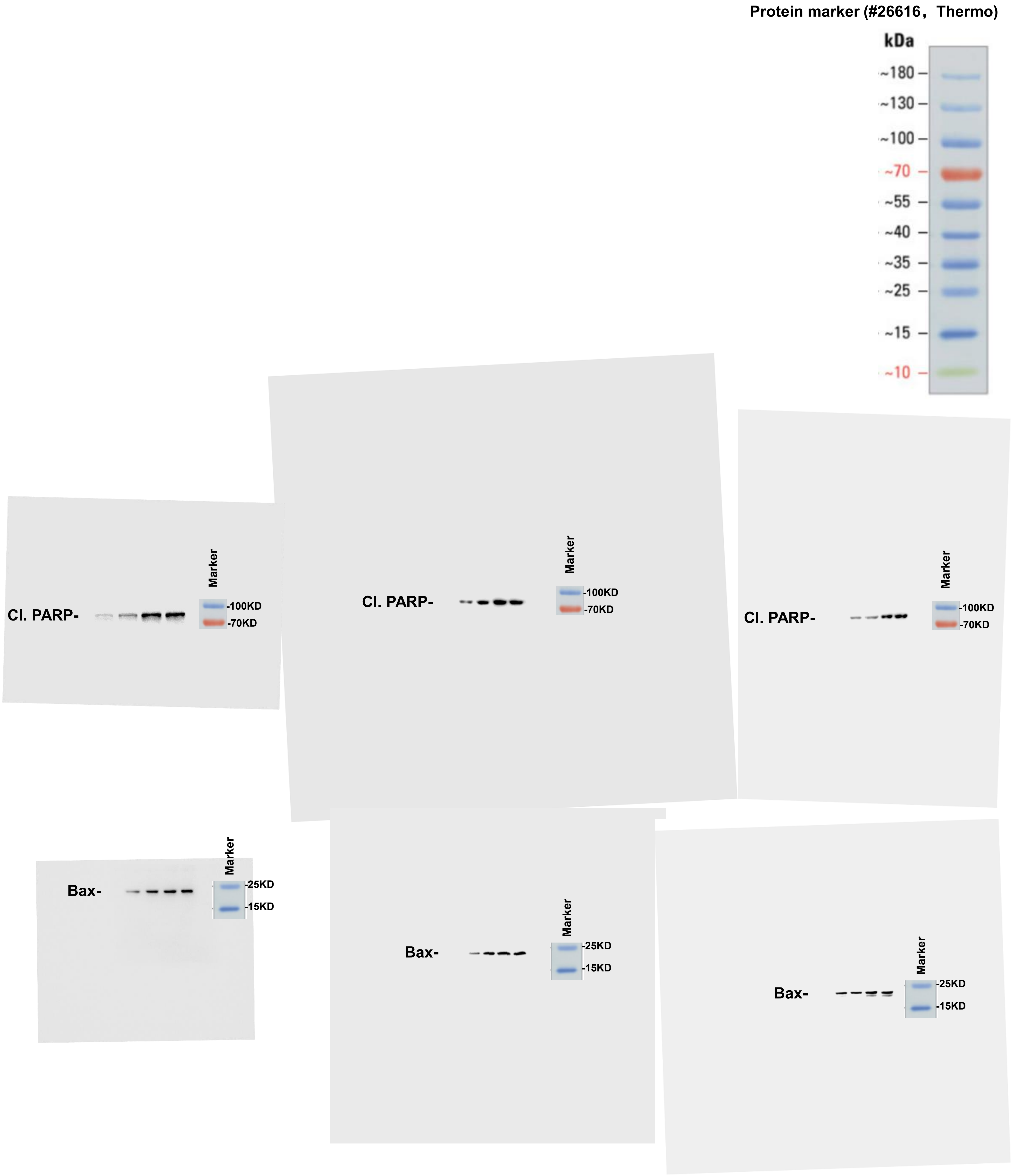

Protein marker (#26616，Thermo)
kDa
~180
~130
~100
~70
~55
~40
~35
~25
~15
~10
Marker
Cl. PARP-
-100KD
-70KD
Marker
Cl. PARP-
-100KD
-70KD
Marker
Cl. PARP-
-100KD
-70KD
Marker
Bax-
-25KD
-15KD
Marker
Bax-
-25KD
-15KD
Marker
Bax-
-25KD
-15KD

Figure 3E

Protein marker (#26616，Thermo)

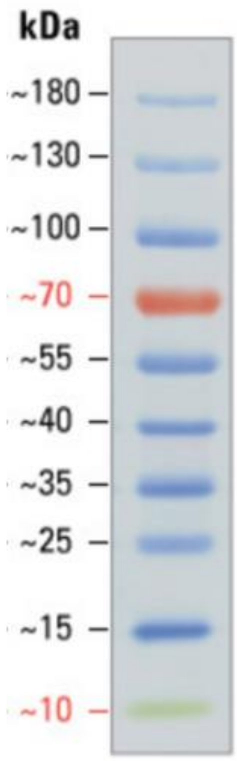

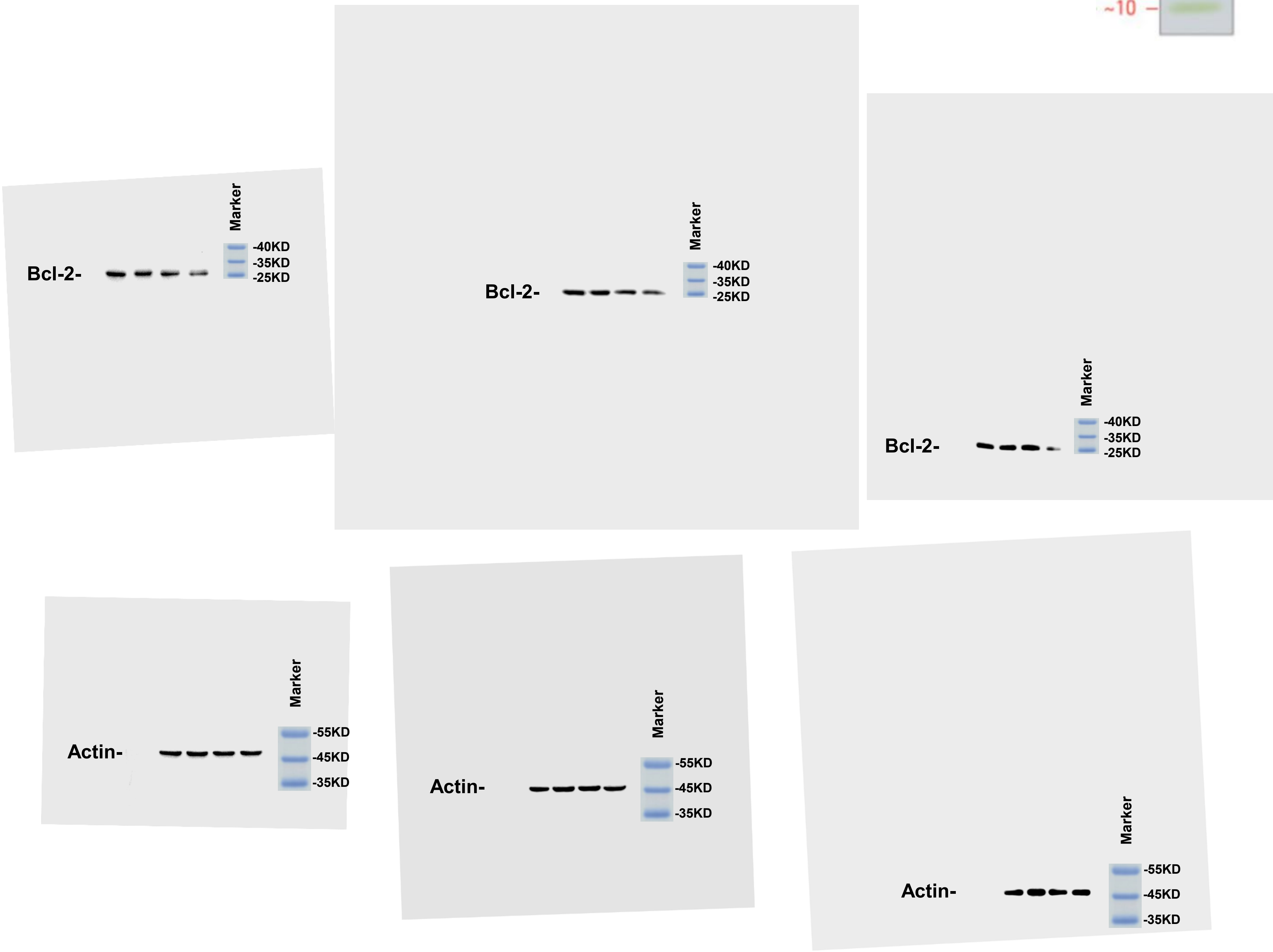

Figure 4A

Protein marker (#26616，Thermo)

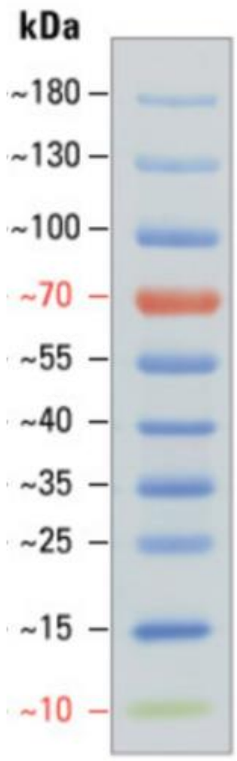

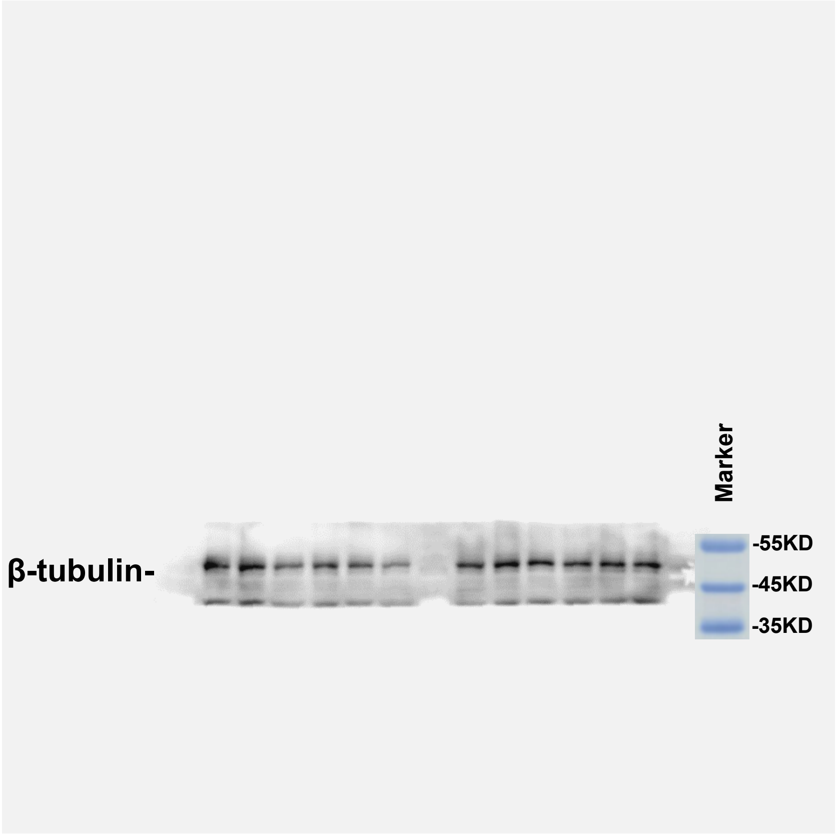

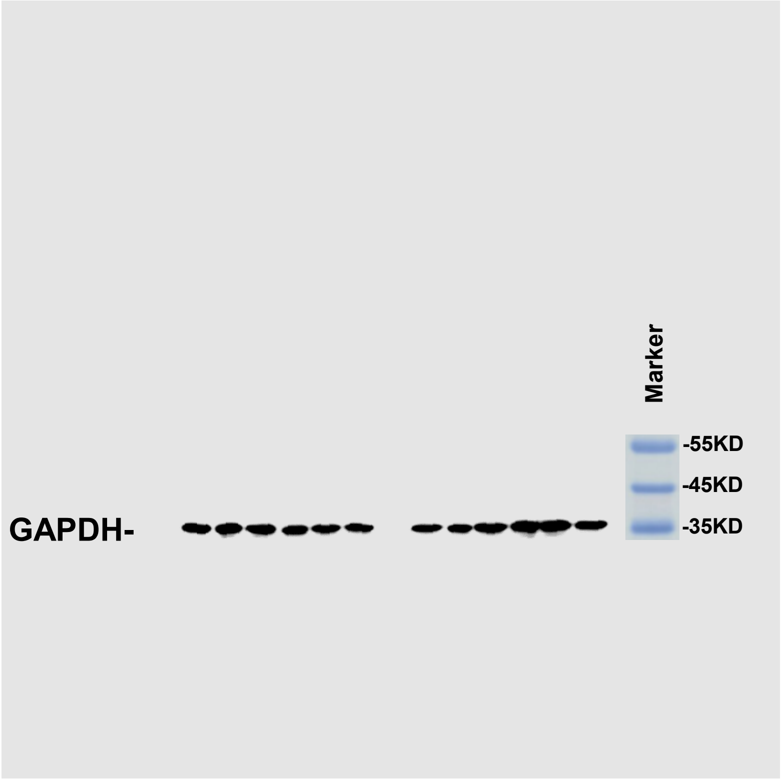

Figure 4C

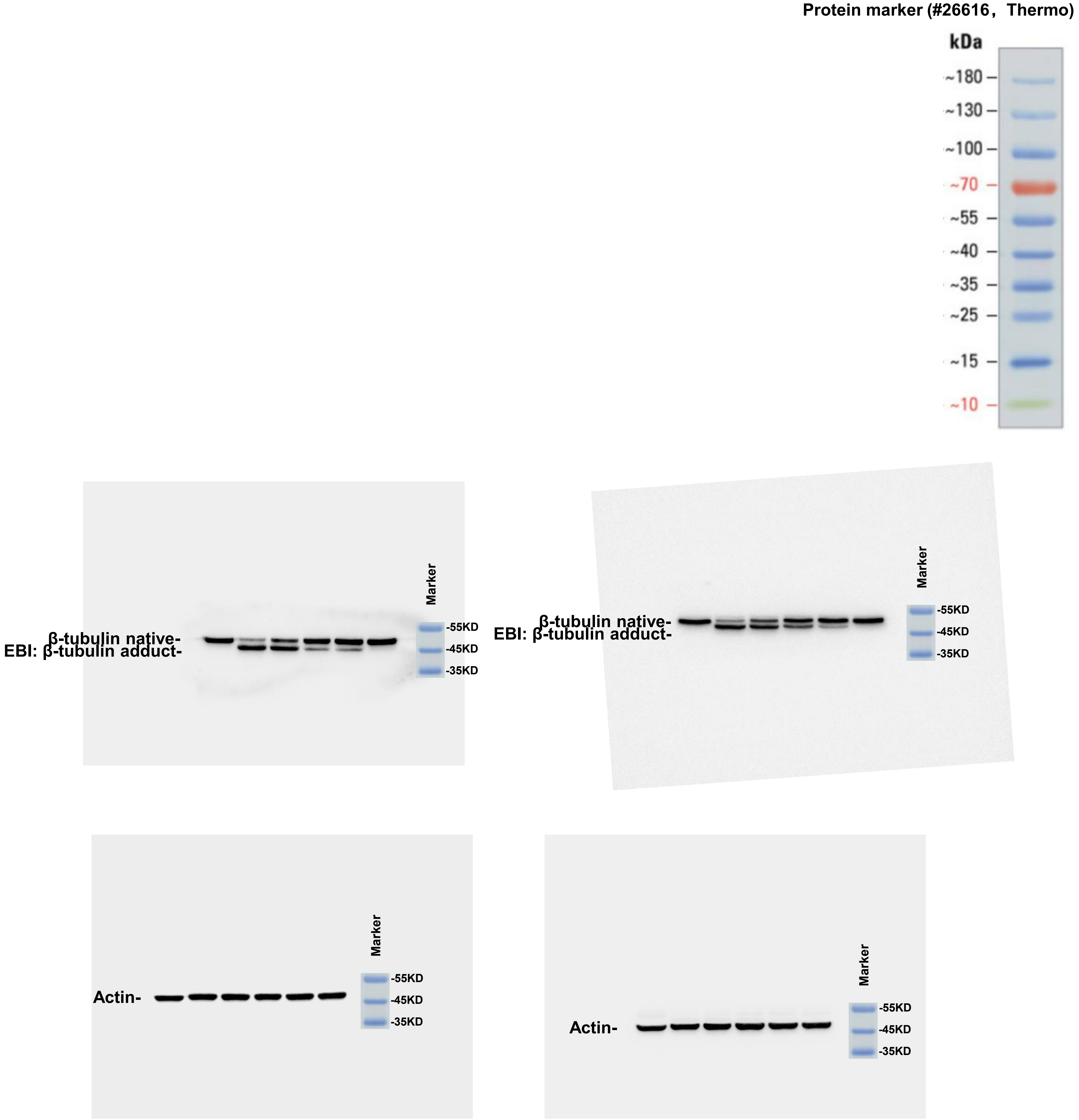
Protein marker (#26616，Thermo)
kDa
~180
~130
~100
~70
~55
~40
~35
~25
~15
~10
Marker
β-tubulin native-
EBI: β-tubulin adduct-
-55KD
-45KD
-35KD
Marker
β-tubulin native-
EBI: β-tubulin adduct-
-55KD
-45KD
-35KD
Marker
Actin-
-55KD
-45KD
-35KD
Marker
Actin-
-55KD
-45KD
-35KD

Figure 5C

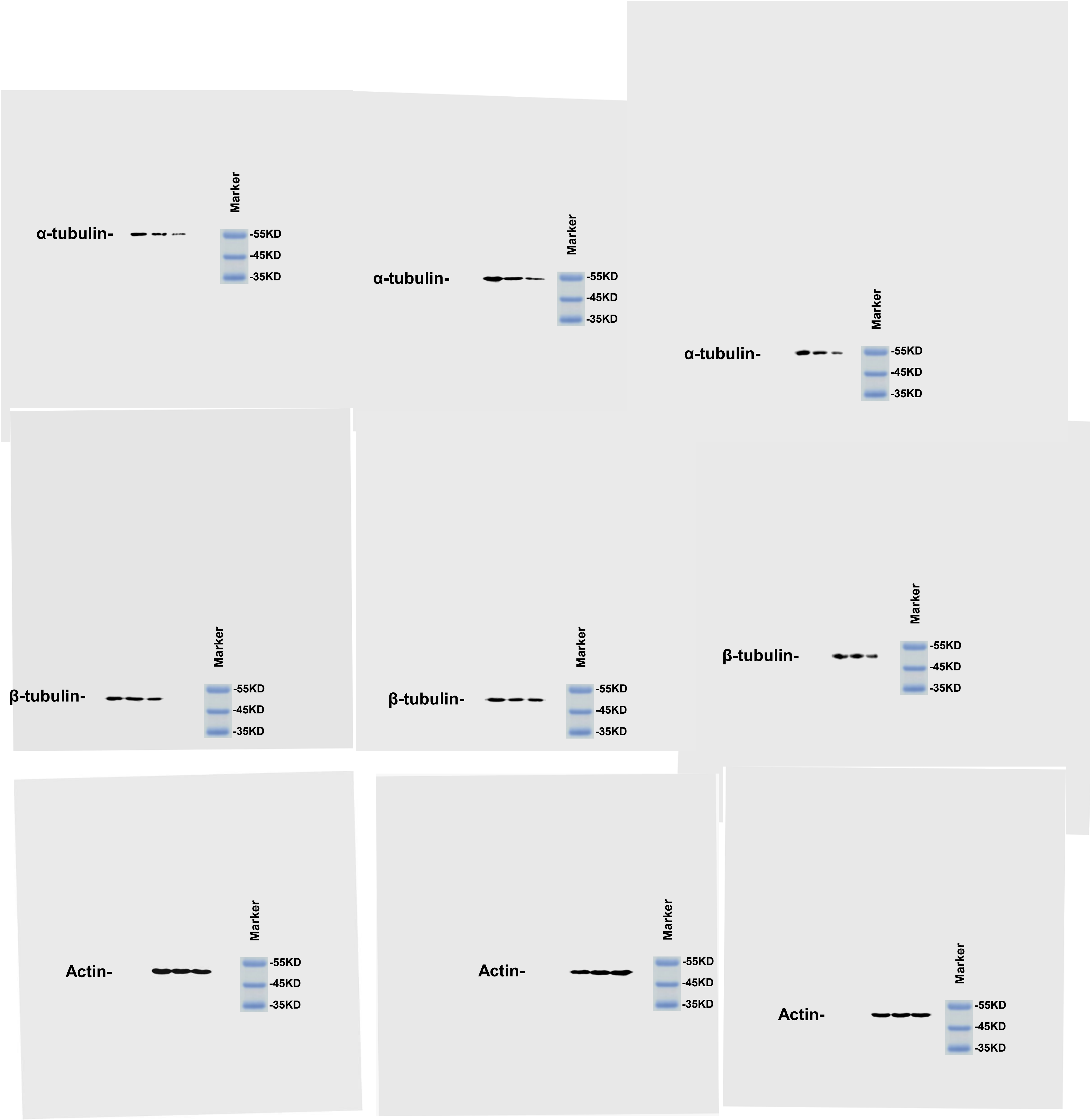

Marker
α-tubulin-
-55KD
-45KD
-35KD
Marker
α-tubulin-
-55KD
-45KD
-35KD
Marker
α-tubulin-
-55KD
-45KD
-35KD
Marker
β-tubulin-
-55KD
-45KD
-35KD
Marker
β-tubulin-
-55KD
-45KD
-35KD
Marker
β-tubulin-
-55KD
-45KD
-35KD
Marker
Actin-
-55KD
-45KD
-35KD
Marker
Actin-
-55KD
-45KD
-35KD
Marker
Actin-
-55KD
-45KD
-35KD

Figure 6A

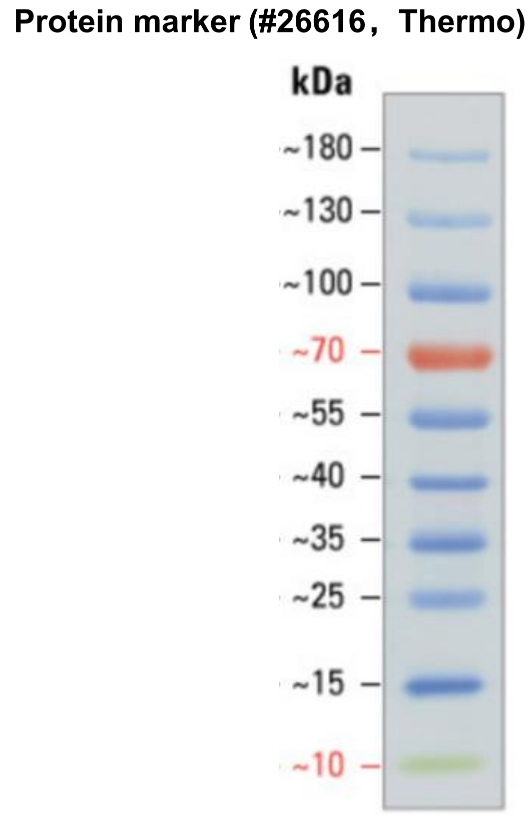
Protein marker (#26616，Thermo)
kDa
~180
~130
~100
~70
~55
~40
~35
~25
~15
~10

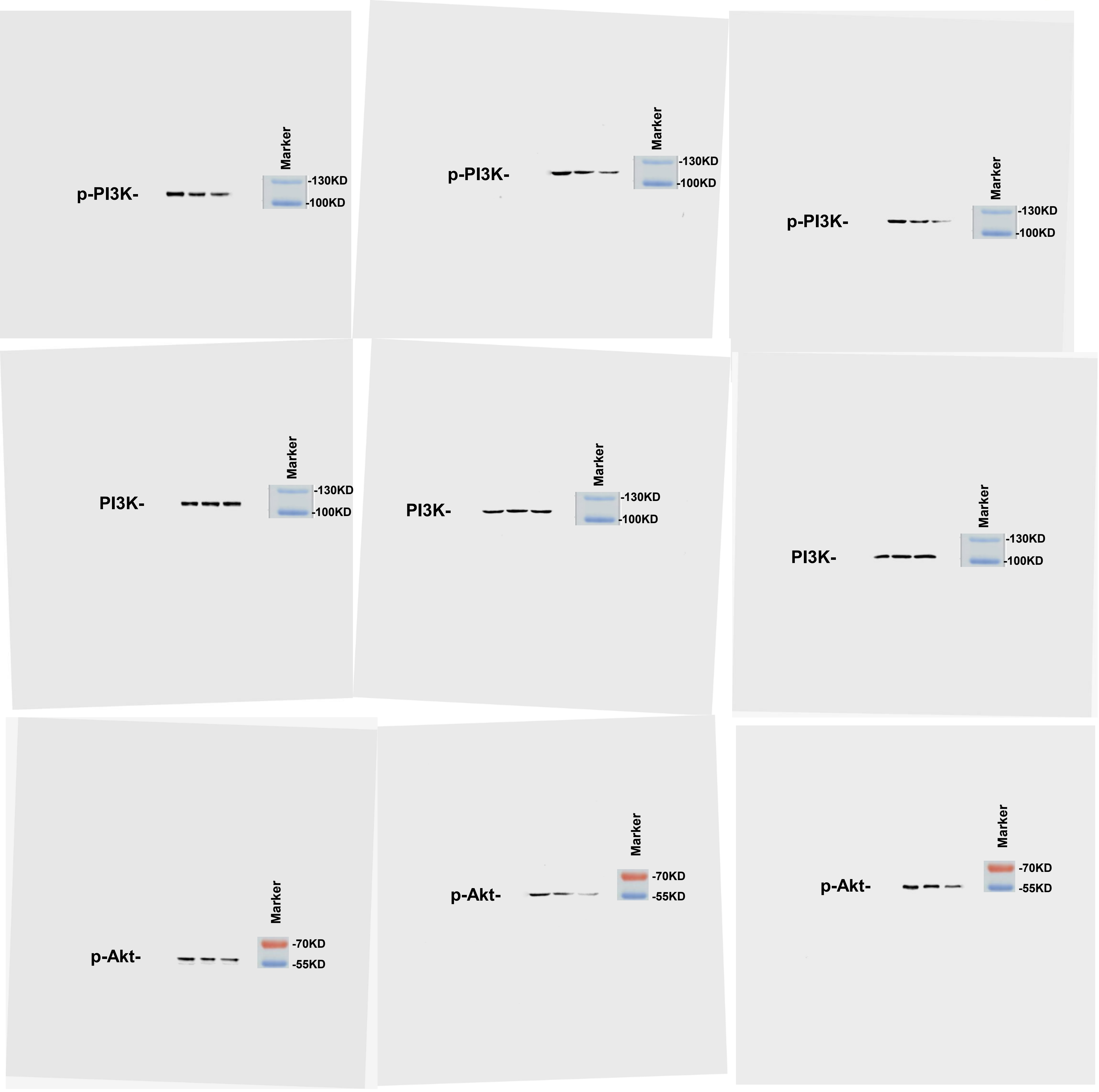
p-PI3K-
Marker
-130KD
-100KD
p-PI3K-
Marker
-130KD
-100KD
p-PI3K-
Marker
-130KD
-100KD
PI3K-
Marker
-130KD
-100KD
PI3K-
Marker
-130KD
-100KD
PI3K-
Marker
-130KD
-100KD
p-Akt-
Marker
-70KD
-55KD
p-Akt-
Marker
-70KD
-55KD
p-Akt-
Marker
-70KD
-55KD

Figure 6A

Protein marker (#26616，Thermo)

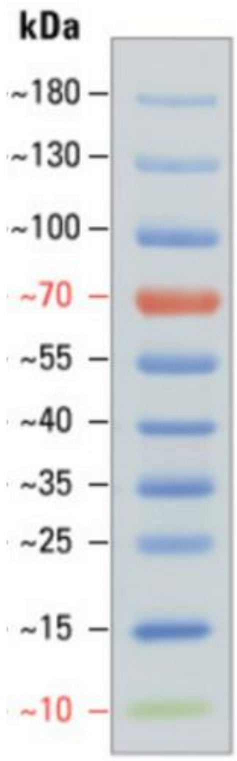

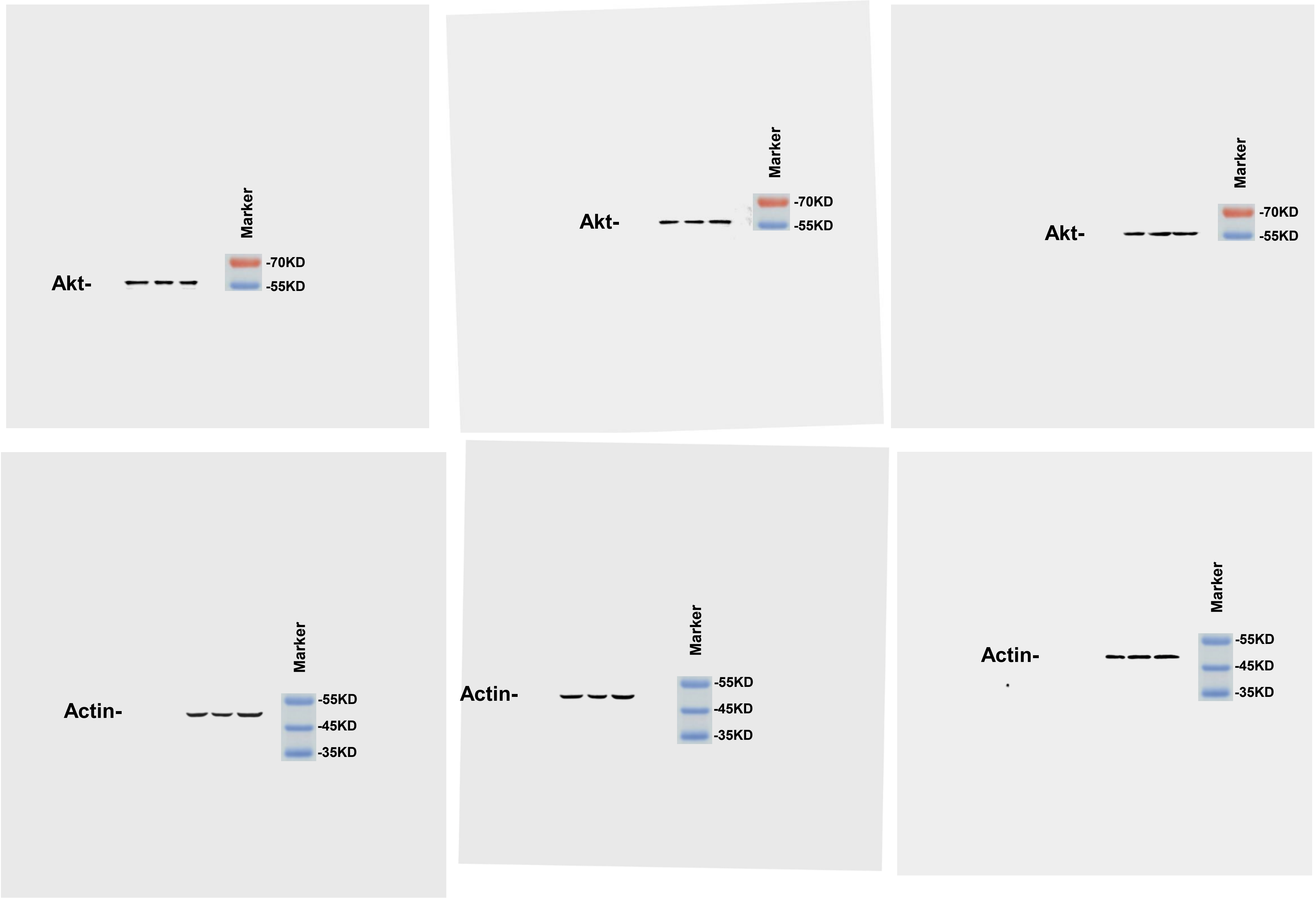

Figure 6D

Protein marker (#26616，Thermo)

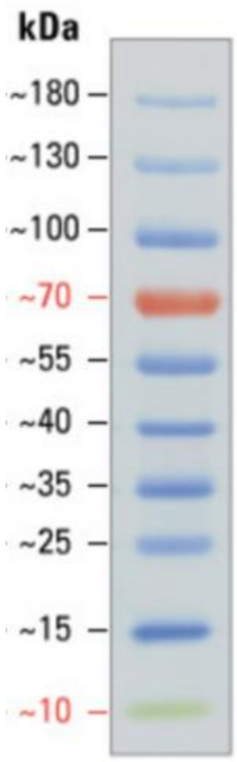

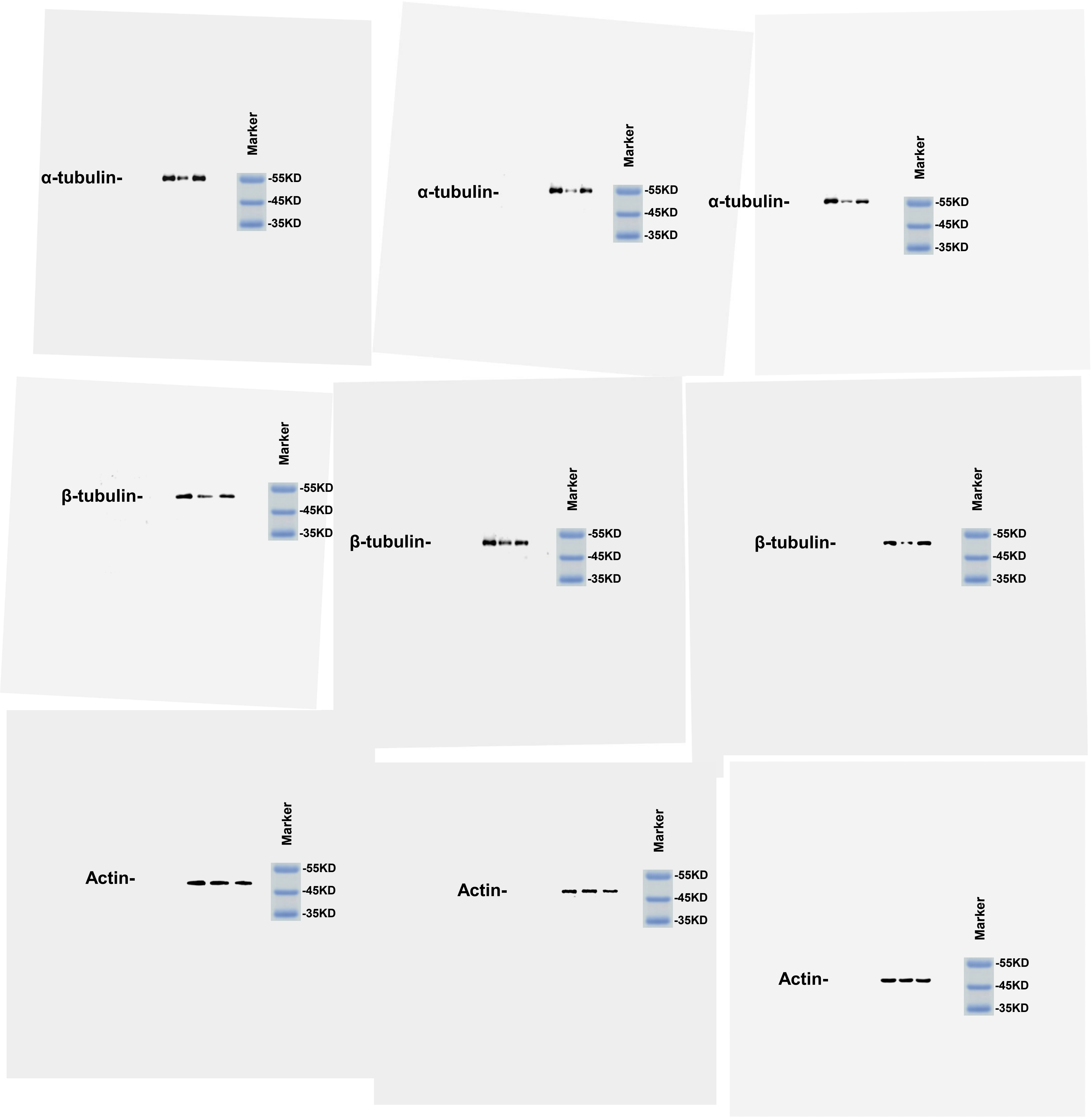

Figure 7J

Protein marker (#26616, Thermo)

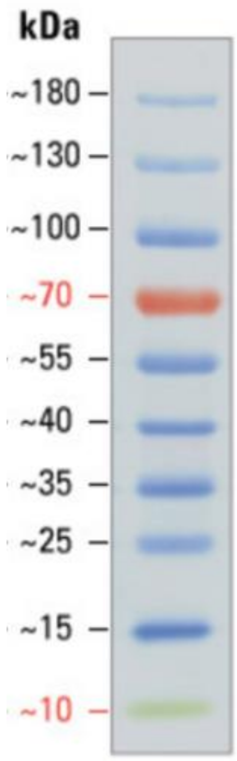

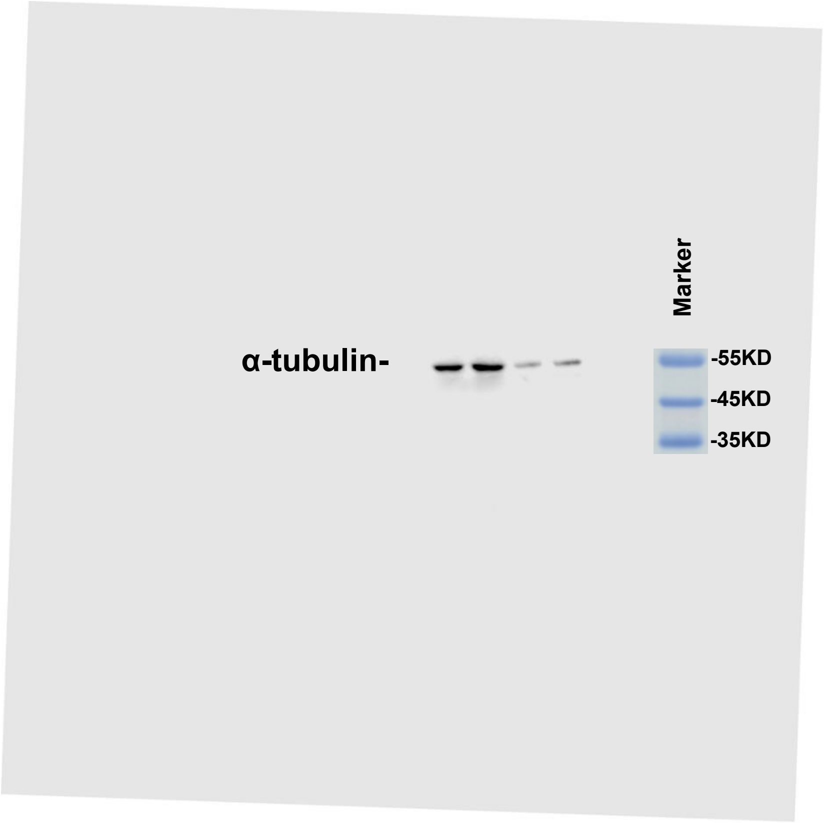

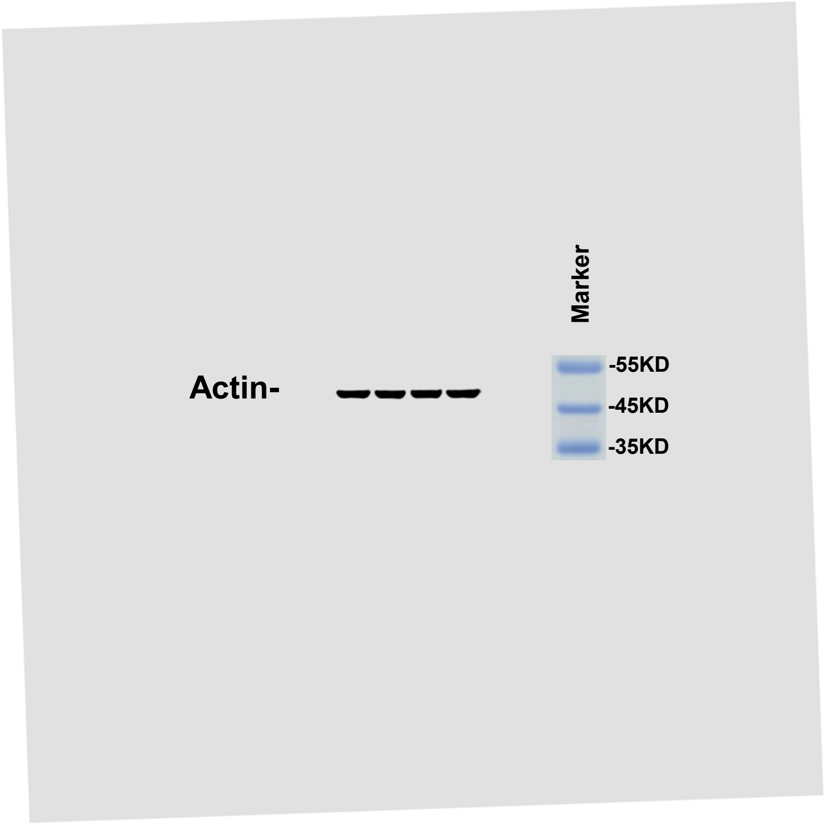

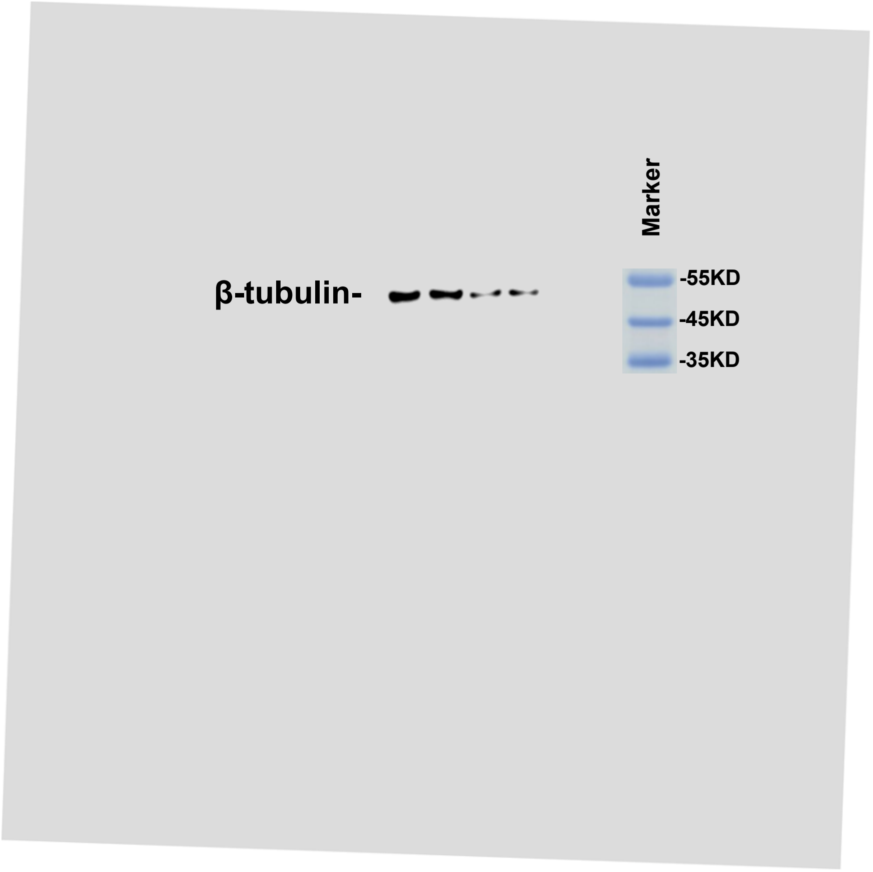

Figure 7K

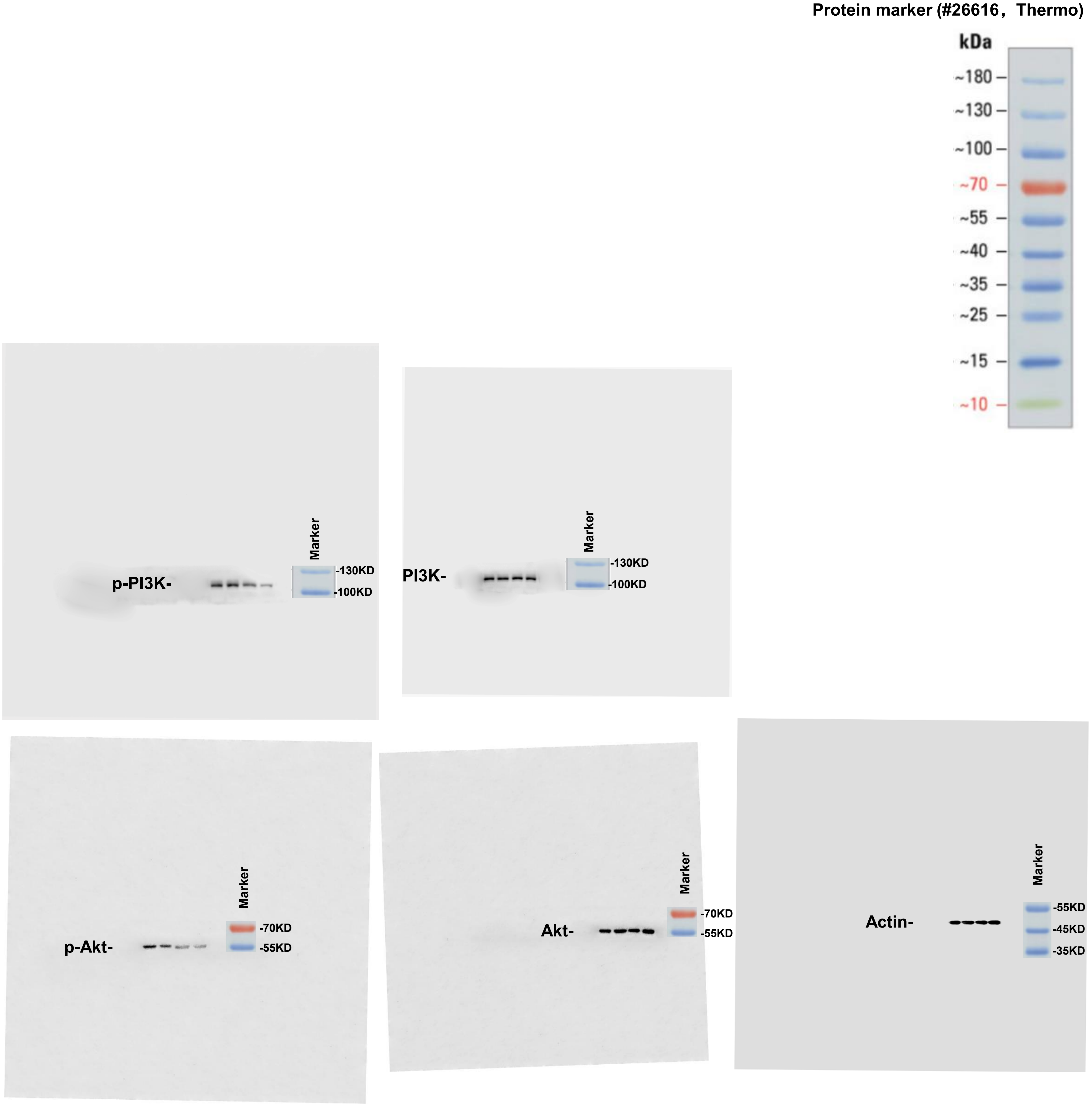
Protein marker (#26616，Thermo)
kDa
~180
~130
~100
~70
~55
~40
~35
~25
~15
~10
p-PI3K-
Marker
-130KD
-100KD
PI3K-
Marker
-130KD
-100KD
p-Akt-
Marker
-70KD
-55KD
Akt-
Marker
-70KD
-55KD
Actin-
Marker
-55KD
-45KD
-35KD
